# Deep Learning for the Radiographic Detection of Periodontal Bone Loss

**DOI:** 10.1038/s41598-019-44839-3

**Published:** 2019-06-11

**Authors:** Joachim Krois, Thomas Ekert, Leonie Meinhold, Tatiana Golla, Basel Kharbot, Agnes Wittemeier, Christof Dörfer, Falk Schwendicke

**Affiliations:** 1 Department of Operative and Preventive Dentistry, Charité - Universitätsmedizin Berlin, Berlin, Germany; 2 CODE University of Applied Science, Berlin, Germany; 3 Clinic for Conservative Dentistry and Periodontology, Christian-Albrechts-Universität Kiel, Kiel, Germany

**Keywords:** Radiography, Panoramic radiography

## Abstract

We applied deep convolutional neural networks (CNNs) to detect periodontal bone loss (PBL) on panoramic dental radiographs. We synthesized a set of 2001 image segments from panoramic radiographs. Our reference test was the measured % of PBL. A deep feed-forward CNN was trained and validated via 10-times repeated group shuffling. Model architectures and hyperparameters were tuned using grid search. The final model was a seven-layer deep neural network, parameterized by a total number of 4,299,651 weights. For comparison, six dentists assessed the image segments for PBL. Averaged over 10 validation folds the mean (SD) classification accuracy of the CNN was 0.81 (0.02). Mean (SD) sensitivity and specificity were 0.81 (0.04), 0.81 (0.05), respectively. The mean (SD) accuracy of the dentists was 0.76 (0.06), but the CNN was not statistically significant superior compared to the examiners (p = 0.067/t-test). Mean sensitivity and specificity of the dentists was 0.92 (0.02) and 0.63 (0.14), respectively. A CNN trained on a limited amount of radiographic image segments showed at least similar discrimination ability as dentists for assessing PBL on panoramic radiographs. Dentists’ diagnostic efforts when using radiographs may be reduced by applying machine-learning based technologies.

## Introduction

Periodontitis is among the most prevalent diseases of humankind, burdening billions of individuals and, if untreated, leading to tooth mobility and, in many cases, tooth loss. To manage periodontitis, early detection and active periodontal therapy as well as systematic and regular supportive periodontal therapy are required. Clinically, detection and diagnosis of periodontal disease builds on assessing probing pocket depths and gingival recessions, resulting in clinical attachment loss measurements. This clinical evaluation, however, has limited reliability, and screening for periodontal disease (for example using the periodontal screening index, PSI) is both a diagnostic effort and likely to miss localized periodontal tissue loss.

A commonly applied additional method to detect and assess the bone loss resulting from periodontal disease is radiography^1^. While bitewing or peri-apical radiographs are often considered as the standard for the assessment of periodontal bone loss (PBL), the radiographic detection of PBL also relies on panoramic scans. Intraoral and panoramic radiographic PBL reading have been found to be largely in agreement with each other^2^, and panoramic readings have been demonstrated to show close correlation with the PSI^3^. Panoramic radiographs may serve to complement and support clinical assessment of PBL.

However, detecting PBL on radiographs is marred by the limited accuracy of individual examiners and the low reliability between different examiners^4^, as demonstrated by a large range of studies and against various reference tests^5^. Hence, calls for automated assistance-systems for dental radiographic imagery data have been raised^6^. Such automated systems could allow more reliable and accurate assessments of PBL, for example on panoramic dental radiographs. For such panoramic images, automated assistance-systems seem especially useful given the high human efforts required for a systematic, comprehensive and reliable assessment.

In healthcare, convolutional neural networks (CNNs) have been successfully applied to detect, for example, breast cancer in mammographies^7^, skin cancer in clinical skin screenings^8^, or diabetic retinopathy in eye examinations^9^. In dentistry, pretrained CNN architectures have been applied to detect carious lesions on bitewing radiographs^10^ or PBL on peri-apical radiographs^11^. However, by applying strict selection criteria for tooth type (excluding anterior teeth), tooth status (heavily restored or root-canal treated teeth were excluded) and quality (only images with high quality were included), the generalizability of the findings of these studies may be limited. Also, no study has been performed to test the application of deep CNNs to radiographically detect PBL on panoramic scans, while the additional diagnostic yield (gained accuracy and reliability, time savings for the assessment) may be highest on such scans given their complexity and the associated diagnostic efforts, as described.

We aimed to apply deep CNNs on dental radiographic imagery to detect PBL on image segments of panoramic dental radiographs. We hypothesized that a CNN showed significantly superior diagnostic performance, measured by classification accuracy, compared to experienced dentists for detecting PBL on radiographic images. The reference test was the measured radiographical PBL (in % of the root length), quantified by three independent examiners. This cutoff value was varied between 20% (base-case) and 30% in sensitivity analyses.

## Results

We used a seven-layer feed-forward CNN with a total number of 4,299,651 trainable weights to predict PBL (see Appendix Fig. S1, Table S1). The base-case model (20% PBL calculated from the three reference test measurements) was trained on average (SD) on 1,456 (47) images and validated on 353 (25) images, respectively. The mean (SD) accuracy of the CNN was 0.81 (0.02). Using a threshold of 0.5, mean (SD) sensitivity and mean specificity was 0.81 (0.04) and 0.81 (0.05), respectively; further metrics are shown in Table 1. The top-ten true and false positively labelled images are shown in the appendix. However, no clear pattern of why predictions were not accurate was identified.

**Table 1:** Base-case and sensitivity analyses.

T.	PBL	Ref. test	Prevalence valid. set	Images train. set	Images valid. set	Acc.	AUC	F1	Sens.	Specif.	PPV	NPV
All*	20%	Average	0.43 ± 0.05	1456.4 ± 44.6	353.2 ± 24.6	0.81 ± 0.02	0.89 ± 0.02	0.78 ± 0.03	0.81 ± 0.04	0.81 ± 0.05	0.76 ± 0.05	0.85 ± 0.02
All	25%	Average	0.28 ± 0.04	1873.0 ± 49.7	353.2 ± 24.6	0.81 ± 0.04	0.88 ± 0.02	0.68 ± 0.05	0.75 ± 0.08	0.82 ± 0.07	0.64 ± 0.10	0.90 ± 0.02
All	30%	Average	0.18 ± 0.05	2185.0 ± 59.6	353.2 ± 24.6	0.81 ± 0.06	0.89 ± 0.02	0.57 ± 0.09	0.72 ± 0.15	0.83 ± 0.10	0.50 ± 0.11	0.94 ± 0.03
in	20%	Average	0.45 ± 0.06	1456.4 ± 44.6	102.0 ± 7.4	0.75 ± 0.03	0.84 ± 0.03	0.73 ± 0.05	0.77 ± 0.07	0.73 ± 0.08	0.70 ± 0.06	0.80 ± 0.03
ca	20%	Average	0.26 ± 0.08	1456.4 ± 44.6	51.7 ± 4.6	0.83 ± 0.04	0.86 ± 0.04	0.63 ± 0.14	0.63 ± 0.17	0.89 ± 0.04	0.65 ± 0.13	0.88 ± 0.05
pm	20%	Average	0.34 ± 0.07	1456.4 ± 44.6	93.3 ± 7.1	0.80 ± 0.04	0.88 ± 0.05	0.73 ± 0.07	0.79 ± 0.07	0.81 ± 0.05	0.68 ± 0.10	0.88 ± 0.04
m	20%	Average	0.56 ± 0.05	1456.4 ± 44.6	106.2 ± 10.3	0.86 ± 0.03	0.94 ± 0.03	0.88 ± 0.03	0.88 ± 0.06	0.84 ± 0.08	0.88 ± 0.06	0.85 ± 0.06

The accuracy, the area-under-the-curve (AUC), the F1-score, sensitivity, specificity and positive/negative predictive values (PPV, NPV) as mean (SD) values are shown. In the base-case (marked by an asterisk *), all teeth were included during training, and the cut-off for periodontal bone loss (PBL) in the reference test was set at 20% of the average of three independent measurements. For sensitivity analyses we varied the cut-offs (increasing them to 25% and 30%), and also validated the performance on specific subsets of teeth. Note that the number of images in the training set varies due to oversampling of the minority (prevalent) class.

T. All: all teeth, in: incisors, c: canine, pm: premolars, m: molars.

Ref. test: Average: Reference test was set at 20% (25%, 30%) of the average of three independent measurements.

**Figure 1:**
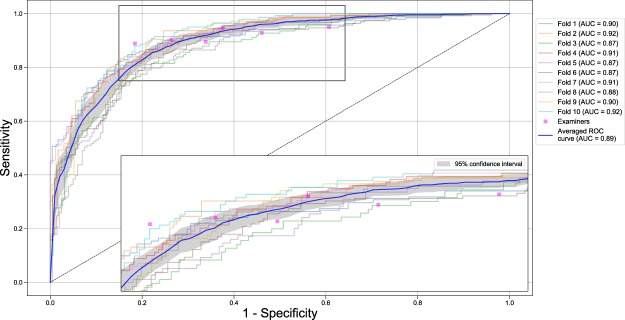
Receiver operating characteristic (ROC) curves for the base-case model (reference test defined by average of three independent measurements of the % PBL, cut-off 20%, all teeth included). The CNN was evaluated against the reference test with respect to sensitivity (the proportion of positives that are correctly identified as such) and specificity (the proportion of negatives that are correctly identified as such). The colored curves indicate the discrimination ability in each validation fold. The bold blue line represents the mean discrimination ability; the gray area corresponds to the 95% confidence interval (CI), respectively. The discrimination ability is further summarized by the AUC. A single examiner’s discrimination ability is represented by a magenta marker (operating point). If the operating point for a particular examiner lies outside the 95% CI, the model’s discriminative ability is significantly different (superior, inferior) from the examiner’s. Insert: magnified area. Sensitivity analyses with variations in reference test construction, cut-off and tooth subsets can be found in Table 1 and the appendix.

The six assessing dentists agreed on their judgment of PBL being present or not on 50.3% images; Fleiss kappa was 0.52, i.e. moderate. The mean (SD) accuracy of the dentists was 0.76 (0.06), but the CNN was not statistically significant superior compared to the examiners (p = 0.067). Further metrics are shown in Table 2. The sensitivity and specificity of the dentists was 0.92 (0.02) and specificity 0.63 (0.14), respectively (Table 2). Figure 1 shows the Receiver Operating Characteristic (ROC) curves for the base-case model and the examiners’ operating point, which allows to graphically compare the discrimination ability of the CNN and the examiners. One examiner showed significantly higher, two a similar and three a significantly lower discriminative ability than the CNN, respectively.

**Table 2:** Performance of the six dentists (mean, SD) on the image segments of the validation datasets.

T.	PBL	Ref. test	Prevalence valid. set	Images valid. set	Acc.	AUC	F1	Sens.	Specif.	PPV	NPV
All*	20%	Average	0.45 ± 0.00	1635.3 ± 4.5	0.76 ± 0.06	0.77 ± 0.06	0.78 ± 0.04	0.92 ± 0.02	0.63 ± 0.14	0.68 ± 0.07	0.90 ± 0.02
All	25%	Average	0.31 ± 0.00	1635.3 ± 4.5	0.66 ± 0.08	0.74 ± 0.05	0.64 ± 0.05	0.95 ± 0.01	0.53 ± 0.12	0.48 ± 0.06	0.96 ± 0.01
All	30%	Average	0.20 ± 0.00	1635.3 ± 4.5	0.56 ± 0.08	0.71 ± 0.05	0.47 ± 0.04	0.96 ± 0.02	0.46 ± 0.10	0.31 ± 0.04	0.98 ± 0.01
in	20%	Average	0.49 ± 0.00	472.5 ± 2.1	0.73 ± 0.08	0.73 ± 0.07	0.77 ± 0.05	0.89 ± 0.04	0.58 ± 0.17	0.68 ± 0.07	0.84 ± 0.04
ca	20%	Average	0.29 ± 0.00	240.7 ± 0.5	0.73 ± 0.09	0.79 ± 0.06	0.67 ± 0.07	0.91 ± 0.04	0.67 ± 0.14	0.54 ± 0.09	0.95 ± 0.02
pm	20%	Average	0.37 ± 0.00	437.2 ± 1.1	0.75 ± 0.07	0.78 ± 0.05	0.73 ± 0.05	0.91 ± 0.03	0.65 ± 0.13	0.62 ± 0.09	0.92 ± 0.02
m	20%	Average	0.58 ± 0.00	485.0 ± 1.2	0.81 ± 0.05	0.79 ± 0.07	0.86 ± 0.03	0.95 ± 0.04	0.62 ± 0.17	0.79 ± 0.08	0.92 ± 0.04

The accuracy, the area-under-the-curve (AUC), the F1-score, sensitivity, specificity and positive/negative predictive values (PPV, NPV) are shown. In the base-case (marked by an asterisk *), all teeth were analyzed, and the cut-off for periodontal bone loss (PBL) in the reference test was set at 20% of the average of three independent measurements. For sensitivity analyses we varied the cut-offs (increasing them to 25% and 30%), and also included only specific subsets of teeth in the analyses.

T. All: all teeth, in: incisors, c: canine, pm: premolars, m: molars.

Ref. test: Average: Reference test was set at 20% (25%, 30%) of the average of three independent measurements.

A number of sensitivity analyses were performed (Tables 1 and 2). (1) By increasing the cut-off value of PBL from 20% to 25% and 30%, performance metrics only limitedly changed. For the dentists, specificity decreased drastically, and with it the accuracy, AUC, F1-score and PPV. Only sensitivity and NPV increased. (2) The CNN was trained on all teeth but validated (applied) only to a particular tooth type, and also the dentists’ performance was only determined on specific tooth types. On molars, both the CNN and the examiners’ performance increased. On premolars and canines, only limited differences to the base-case were detected. On incisors, the performance decreased.

## Discussion

The present study assessed the diagnostic performance of a CNN for detecting PBL, and compared it to six experienced dentists. We assumed a CNN to be useful, yielding higher accuracy and reliability than dentists for detecting PBL while decreasing diagnostic efforts by saving time and reducing documentation. This may allow for more targeted subsequent clinical or radiographic diagnostic (using bitewings or peri-apical images) and treatment. A number of analyses explored factors impacting on the performance of both the CNN and examiners. Our hypothesis was that a CNN would perform better, measured via the classification accuracy, than the dentists. We reject that hypothesis, as we could not confirm significantly better performance. Our findings also highlight the limited agreement between the dentists.

The applicability and accuracy of CNNs may be further improved when additional imagery data (e.g. peri-apicals) or data sources (clinical records) are integrated into the analytic pipeline. Also, further assessment of the PBL morphology (horizontal or vertical bone loss) and other radiographically assessable items (root morphology) may be available using CNNs. The integration of these data could allow automated prediction making using algorithms^12^.

Our CNN showed lower sensitivity than individual examiners, especially when a higher cut-off definition for PBL was used. In practice, a CNN may be used mainly to highlight likely PBL, requiring high sensitivity (note the risk of over-detection and over-treatment associated with high sensitivity). It remains unclear why the model was consistently more specific than examiners. Visualizing what image features are operationalized by the CNN to come to a decision may enhance our understanding behind that observation. Inspecting images which were false positively or negatively classified, as shown in the appendix, was not found to provide such deeper understanding.

We applied a straightforward custom-made model architecture. We had, during our model development phase, attempted to leverage pretrained CNNs, such as VGG16 and Inception.v3. However, we found that these architectures caused overfitting and reduced the discrimination ability. In comparison with other studies in the field, we had very broad inclusion criteria towards image quality, tooth type and tooth status, as discussed. This may be the reason why needed to construct a custom-made CNN, while other studies (with narrower inclusion criteria) leveraged pretrained networks despite using a similar number of images^11^. Notably, though, our CNN yielded higher accuracies when compared to the reported accuracies of pretrained CNNs. Especially on image subsets which seemed easier to assess (e.g. molars or images with reference test agreement), AUC values of 0.94–0.95 were found, by far outperforming reported accuracies of pretrained models. We had actively decided against narrow inclusion criteria, as one relevant task of a CNN may be to direct a dentist’s diagnostic effort towards images which are hard to assess. Future studies should investigate the impact of factors like image projection or quality contrast on the discrimination ability, and aim to improve the model performance accordingly. A triangulation of radiographic findings with clinical assessments should be considered, too.

Increasing the cut-off for the reference tests had a remarkable impact. While the accuracy of the CNN was not severely affected, that of the dental examiners decreased significantly, mainly as the specificity decreased while the increase in sensitivity could not compensate for this. The relevance of this on the subsequent decision-making in practice should be explored.

This study has a number of limitations. First, in the realm of computer vision and deep learning, our dataset can be considered small. To improve the model’s performance, larger datasets will be needed. More image instances and triangulated, robust labeling may allow to leverage transfer learning, as described. Second, we assessed manually cropped image segments from panoramic radiographs. Assessing the whole dentition instead may improve the accuracy, as most oral conditions (PBL, caries) are known to be clustered (correlated) within one mouth^13^. Using segmentation algorithms first and then applying CNNs for detecting PBL on image segments while retaining the information of clustering may be a further, worthwhile technical direction. Third, and as discussed, using panoramic scans for PBL detection remains controversial; we accepted this caveat given the expected high diagnostic yield of CNNs especially on complex imaging material like panoramic compared with peri-apical radiographs. Last, cross-validation was performed, without a fully independent hold-out test set. This approach was applied for hyperparameter tuning and for the sensitivity analyses, accounting for the limited imagery material. Hence, it is conceivable that the generalization performance is not fully unbiased due to data leakage. Future studies should, using larger datasets, work with a fully independent hold-out test dataset in order to establish a less biased generalization performance. Alternatively, nested cross-validation would have been an option, which however is computational expensive^14^.

In conclusion and within the limitations of this study, a moderately complex CNN trained on a limited amount of labeled radiographic images showed at least similar diagnostic performance as experienced dentists to detect PBL. A range of aspects showed relevant impact on this performance and should be carefully considered by future studies. The application of CNNs seems promising for assisting dentists with dental imagery diagnostics.

## Materials and Methods

### Study design

This study used a dataset of segmented panoramic radiographs. The reference test was the measured radiographically PBL (in % of the root length), quantified by three independent examiners. A deep CNN was constructed to detect PBL on the radiographs. We compared the performance of the CNNs against the subjective assessments by six dental practitioners. Reporting of this study follows the STARD guideline^15^.

### Performance metrics

Our primary performance metric was classification accuracy, which corresponds to the proportion of correct classifications per all classifications made. Secondary metrics were the AUC, which relates to a classifier’s (a model’s, an examiner’s) ability to avoid false classification, the F1-score which accounts for the relations between data’s positive labels and those given by a classifier, sensitivity and specificity, and the positive and negative predictive values. Details on the performance metrics are provided in the appendix.

### Sample size calculation

Based on our hypothesis, we estimated the minimally required sample size allowing to detect significant differences in the accuracy between the two index tests, i.e. the CNN and the experienced dentists, when both assessed the same subjects (radiographs). Sample size calculation was based on the assumption that dentists would, in mean, show an accuracy of 0.80 to detect PBL^11^. We aimed to capture an accuracy-difference between dentists and the CNN of 0.03 (assuming the CNN to have an accuracy of 0.83 in mean) using a t-test (see below). Standard deviations were assumed to be 0.10 in both groups. At α = 0.05 and requiring an 80% power resulted in a sample size of n = 350 images (G* Power 3.1.9.2, Universität Düsseldorf, Germany). As we planned to use the majority of image segments for training, not validation, we eventually aimed to work with 1750 image segments; 350 for validation and 1400 for training.

### Image Dataset

We synthesized a dataset of 2001 manually cropped image segments, each focusing on one particular tooth, from 85 randomly chosen digital panoramic dental radiographs collected using Orthophos XG 3 (Sirona, Bensheim, Germany, year of construction: 2009) according to manufacturer’s instructions (considering patients sex and age etc.). Data collection was ethically approved (Charité ethics committee EA4/080/18); the ethics committee waivered the need for an informed consent given data being pseudonymized. Details on the imaging process can be found in the appendix. Only radiographs from dentate individuals were included. Prior to image processing, one examiner pre-screened the resulting tooth segments, and 264 tooth segments (mainly on incisors) were excluded, most of them as they heavily overlapped with the vertebrae and did not allow any kind of assessment (examples excluded and included images are provided in the appendix). No further in- or exclusion criteria were applied, i.e. the quality of the panoramic images (contrast, hazing, positioning etc.) was not used to exclude images (as we assumed a CNN needed to be able to detect PBL on possibly suboptimal images to be useful in the real world, or to highlight diagnostic uncertainty).

### Reference test

Our reference test was the maximal radiographically detectable PBL in % of the root length. For each tooth, three examiners independently and manually determined three points on each radiograph to estimate PBL in %; the mesial and distal cemento-enamel junction (CEJ), the deepest point of the root apex (for multi-rooted teeth, of the mesial and distal root) and the most apical extension of the alveolar crest (for multi-rooted teeth, the deepest extension mesial and distal was considered). If the CEJ was covered by a restoration, the most apical point of the restoration was used instead^16^. Using these points, it was now possible to calculate the % of PBL as the distance between the CEJ and the alveolar crest divided by the distance of the CEJ to the apex. For multi-rooted teeth, two % (one for the mesial and one for the distal root) were available and only the larger % recorded. Using the % PBL and not the absolute measures (in mm etc.) helps to overcome the described issue of patient positioning and magnification. The result of these three independent measurements were three %-value of PBL for each tooth segment (Fig. S2).

Details on these three measurements can be found in the appendix. In our base-case analysis, we first calculated the mean of these three measurements and then applied a cut-off value of 20%^17^ to distinguish between PBL being present (≥20%) or not (<20%). In a sensitivity analysis, we increased this cut-off to 25% and 30%, respectively, to account for the variability in PBL definitions, but also the different ease of detection of more or less severe PBL.

Both the measurement of the reference test as well as the examination of the radiographs by dentists (see below) were performed in Sidexis 4 (Sirona) using the length measurement tool. Measurements and examinations were performed in dimly lit rooms on diagnostic screens and standardized conditions. Both measurements and examinations allowed magnification and enhancement (contrast etc.) tools to be used.

### Modelling via CNNs

A prominent use case of CNNs is to map an input (image) to an output (classification), based on a set of weights, learned from data. CNNs are composed of chained functions, often referred to as layers. Information is passed forward through the network layers to the final (output) layer, and thereby processed by applying intermediate computations^18^. CNNs are specialized kinds of neural networks, which use a mathematical operation called convolution that allows the CNN to extract features from image data. Stacked CNN layers identify different aspects of the input image such as edges, corners and spots or increasingly complex aspects of the image, such as shapes, structures and patterns. Finally, the last few network layers typically are able to take the feature-filtered images and translate them into votes, in our case a binary vote, of PBL being present or not.

The image data was digitally preprocessed: (1) Each image segment was transformed to gray-scale; (2) segments from the upper jaw were flipped by 180 degrees so that in all images, the crowns faced upwards and roots downwards; (3) all pixel values of each image segment were normalized to a fixed range [0, 1]; (4) all image segments we resized to 64 × 64 pixels. Further image augmentation techniques such as rotation, shearing and zooming were applied during CNN training. Data processing was performed using Python, and third party libraries such as NumPy, pandas, scikit-image and scikit-learn.

The dataset was randomly and repeatedly split into training and validation sets by applying group shuffling. An example can be found in the appendix (Table S2). 10-fold repetition of split and model training and validation was performed to evaluate the robustness of the CNN performance. Owing to the small data set size we did not evaluate the model on a hold-out test set. For each split, image segments of one panoramic radiograph (i.e., patient) were kept together either in the training set or in the validation set to prevent training and validation within the same patient. We further oversampled image instances from the minority class (in our case, these were images where PBL was present according to the reference test) to reduce the detrimental effect of class imbalance on model performance^19^. Accordingly, the prevalence of positive class in the training set was close to 0.5. We did not oversample the minority class during validation (Table 1).

CNNs were developed using the TensorFlow framework and Keras. We combined convolutional layers with activation functions (rectified linear units) and max-pooling layers. For the purpose of model regularization we interlaced these sequences with batch-normalization^20^ and dropout layers. As final model layers we used a series of fully connected dense layers and a softmax classifier. Details on the model development are provided in the appendix.

Hyperparameters were systematically tuned via grid search^21^. We considered the number and ordering of stacked layers, the number of units in the hidden layers, the number of convolutional filters, the kernel sizes and activation functions, image preprocessing, different types of optimizers and learning rates, the usage of dropout and batch normalization layers and their parameterizations and positions, the batch size, and image augmentation parameters as hyperparameters. Details on the hyperparameter tuning and image augmentation can be found in the appendix.

### Subjective assessment

The performance of the CNNs against a reference test is only limitedly useful for interpretation. Hence, six experienced dentists (mean (SD; range) clinical experience 6 (3; 3–10 years) additionally assessed the dataset for radiographically detectable PBL (binary outcome). The dentists were either specialists in operative dentistry and periodontology (n = 1), specialized in endodontics (n = 1) or general dentists (n = 4). All worked full-time at a university hospital; three of them had worked in private practice before. Dentists were informed about the background of the study and the diagnostic task, and instructed to decide if, according to their professional opinion, PBL was present or not. We computed the operating point (sensitivity vs. 1-specificity) for each dentist in order to evaluate the examiner’s discrimination ability, and calculated a mean (SD) AUC^22^. A one-sided two-sample Welch’s t-test was used to compare the dentists’ and the CNN’s accuracy, with α = 0.05 as level of significance. In order to assess the inter-rater reliability we computed Fleiss kappa^23^, assuming 0–0.20 as slight, 0.21–0.40 as fair, 0.41–0.60 as moderate, 0.61–0.80 as substantial, and 0.81–1 as almost perfect agreement^24^. Dentists did not revisit their records, and intra-rater reliability was not assessed.

### Sensitivity analyses

A range of sensitivity analyses were performed. First, we applied the reference test cut-offs at 25% and 30% PBL. Second, we systematically evaluated the model’s discrimination ability on different tooth types (molars, premolars, canines, incisors), the rationale being that owing to the radiographic image generation process, anterior teeth are usually more difficult to assess than posterior teeth.

### Ethical approval and informed consent

All experiments were carried out in accordance with relevant guidelines and regulations. Data collection was ethically approved (Charité ethics committee EA4/080/18).

## Supplementary information

Appendix

## Data Availability

Data used in this study can be made available if needed within data protection regulation boundaries.
