# Supplementary material for: Deep Learning for the Radiographic Detection of Periodontal Bone Loss
Source: Sci Rep. 2019 Jun 11;9:8495. doi: 10.1038/s41598-019-44839-3 (PMC6560098; doi:10.1038/s41598-019-44839-3)
Supplement: Supplementary file 1 — Appendix [file 41598_2019_44839_MOESM1_ESM.docx]

**Deep Learning for the Radiographic Detection of Periodontal Bone Loss**

Joachim Krois^1^, Thomas Ekert^1,2^, Leonie Meinhold^1^, Tatiana Golla^1^, Basel Kharbot^1^, Agnes Wittemeier^1^, Christof Dörfer^3^, Falk Schwendicke^1^ *

^1^ Department of Operative and Preventive Dentistry, Charité - Universitätsmedizin Berlin, Germany

^2^ CODE University of Applied Science, Berlin, Germany

^3^ Clinic for Conservative Dentistry and Periodontology, Christian-Albrechts-Universität Kiel, Kiel, Germany

**Appendix**

***Performance metrics***

We evaluated the inter-rater reliability by computing Fleiss’ kappa (1), which is an extension of Scott’s pi (Scott 1955) and assesses the reliability of agreement of nominal-scale ratings among more than two examiners (Fleiss 1971). Hence, it is a measure for within-group reliability.

$\kappa_{Fleiss}=\frac{P^{*}-P_{e}^{*}}{1-P_{e}^{*}}$ (1)

The terms $P^{*}$ and $P_{e}^{*}$ are the overall agreement probability and the probability of agreement due to chance, respectively (Gwet 2008).

We used different model performance metrics such as the area under the receiver operating characteristic curve (AUC), accuracy (2), sensitivity (3), specificity (4), also referred to as recall, the positive (PPV also referred to as precision – 5) and negative (NPV – 6) predictive value, and the F1-score (7), which is the harmonic mean of precision and recall (Sokolova and Lapalme 2009).

$accuracy=\frac{TP+TN}{TP+FP+TN+FN}$ (2)

$sensitivity (recall)=\frac{TP}{TP+FN}$ (3)

$specificity=\frac{TN}{TN+FP}$ (4)

$PPV (precision)=\frac{TP}{TP+FP}$ (5)

$NPV=\frac{TN}{TN+FN}$ (6)

$F1 score=2\times\frac{precision\times recall}{precision+recall}$ (7)

where $TP$ and $TN$ denotes true positive and true negative classiﬁcations, and $FP$ and $FN$ refer to false positive and false negative classiﬁcations, respectively. The AUC relates to a classiﬁer’s ability to avoid false classiﬁcation. The accuracy is the ratio of correct classifications ($TP$*+*$TN$) and the total of all samples. The sensitivity (recall) accounts for the classiﬁer’s ability to identify positive labels, speciﬁcity accounts for the classiﬁer’s ability to identify negative labels, The PPV (precision) accounts for the class agreement of the data labels with the positive labels given by the classiﬁer whereas the NPV accounts for the class agreement of the data labels with the negative labels given by the classiﬁer. The F1-score accounts for the relations between data’s positive labels and those given by a classifier (Sokolova and Lapalme 2009).

***Radiographic imaging process***

Panoramic radiographs had been taken in the central radiographic unit of the Charité – Universitätsmedizin dental center; all dental patients regardless of the department managing them have their radiographs taken there. The timeframe (2010-2017) was chosen as it captured the period where the used radiographic device was in use, and we saw no reason of restricting the timeframe. Positioning was performed according to the manufacturer’s instruction by experienced medical-technical assistants specialized in dental radiography.

The median (minimum/maximum) age of the 85 patients was 51 (15/91) years. The mean (minimum/maximum) number of teeth per patient (i.e. per panoramic radiograph) was 25 (2/32). There were 29, 15, 27 and 29% incisors, canines, premolars and molars, respectively, in the dataset

***Samples for excluded images***


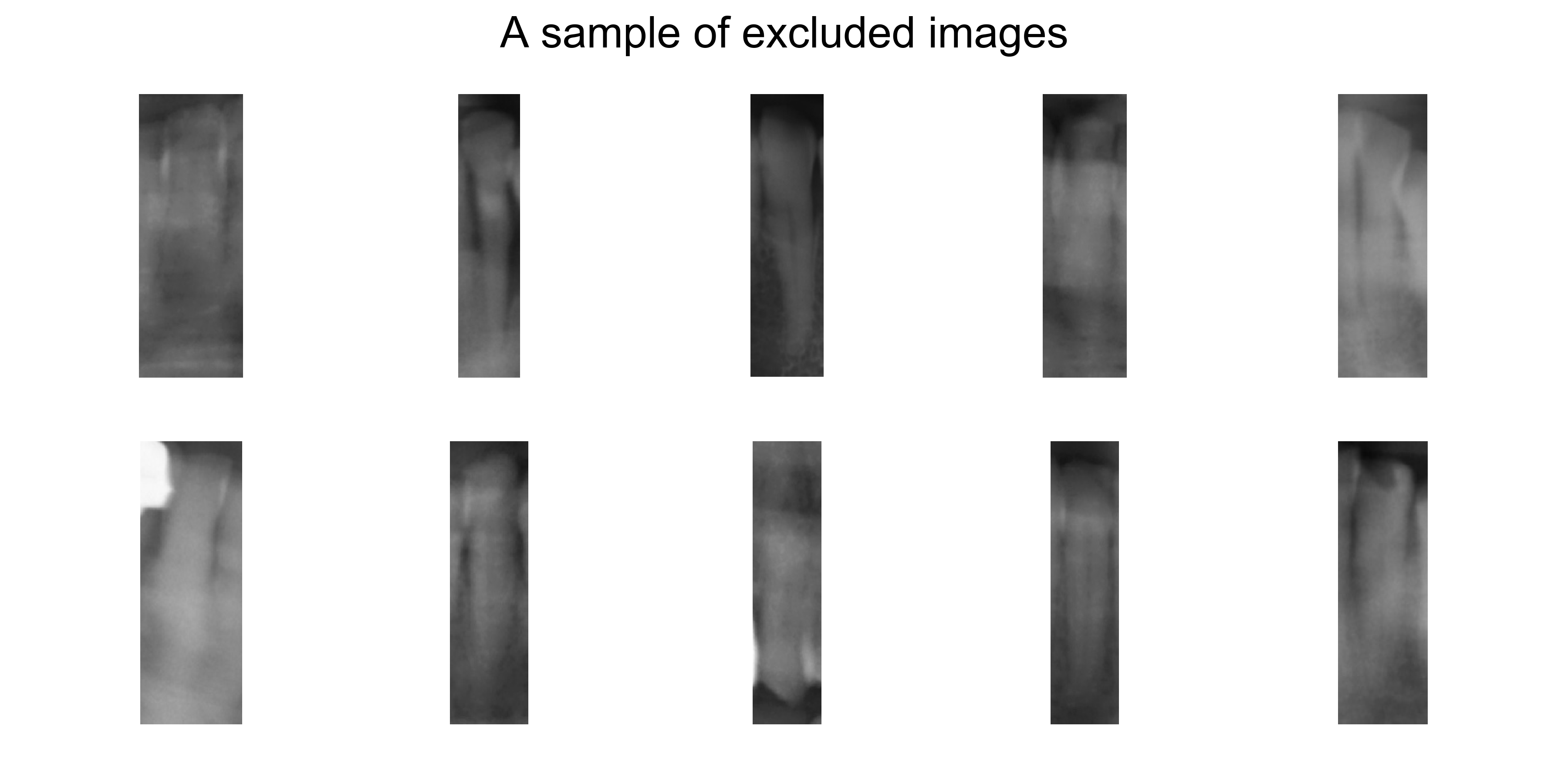


***Reference test***

As described, three independent measurements of the % PBL were performed (Fig. S2). These showed high agreement at 20% (Fleiss Kappa 0.85), 25% (0.81) and 30% (0.81) cut-off values. In the vast majority of images, all three measurements agreed with each other (72-76% depending on the cut-off).


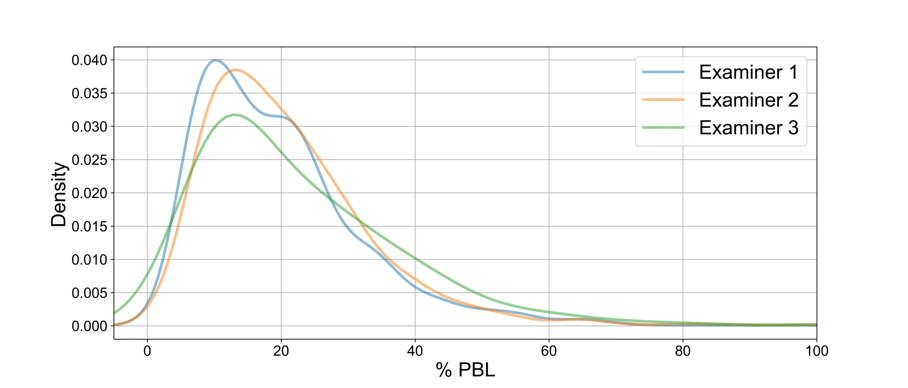
**Figure S2:** Kernel density plot for PBL in %, determined by three independent examiners

***Model parameters***

Table S1: Model layers and hyper parameters.

| **Layer count** | **Layer type** | **Output shape in final model** | **Kernel / Pooling size in final model** | **No. of trainable weights in final model** |
| --- | --- | --- | --- | --- |
|  | Input | 64, 64, 1 |  | 0 |
| 1 | Conv2D | 64, 64, 512 | 3, 3 | 5120 |
|  | ReLU | 64, 64, 512 | - | 0 |
|  | Max Pooling | 32, 32, 512 | 2, 2 | 0 |
| 2 | Conv2D | 32, 32, 256 | 3, 3 | 1179904 |
|  | ReLU | 32, 32, 256 | - | 0 |
|  | Max Pooling | 16, 16, 256 | 2, 2 | 0 |
| 3 | Conv2D | 16, 16, 256 | 3, 3 | 590080 |
|  | ReLU | 16, 16, 256 | - | 0 |
|  | Max Pooling | 8, 8, 256 | 2, 2 | 0 |
| 4 | Conv2D | 8, 8, 128 | 3, 3 | 295040 |
|  | ReLU | 8, 8 128 | - | 0 |
|  | Max Pooling | 4, 4, 128 | 2, 2 | 0 |
|  | Flatten | 2048 | - | 0 |
| 5 | Dense | 1024 |  | 2098176 |
|  | ReLU | 1024 |  | 0 |
|  | DropOut (0.5) | 1024 | - | 0 |
| 6 | Dense | 128 |  | 131200 |
|  | ReLU | 128 |  | 0 |
|  | DropOut (0.7) | 128 | - | 0 |
| 7 | Dense | 1 |  | 129 |
|  | Batch  normalization | 1 | - | 4 |
|  | Softmax | 1 | - | 0 |
|  | DropOut (0.5) | 1 | - | 0 |
|  | Output | 1 | - | 0 |

***CNN Model Architecture***

The base case model was a seven layer CNN with 4,299,651 trainable weights. Its visual representation is shown in Figure S1 and details are given in Table S2. The model architecture was determined using grid search. We evaluated the performance of each particular architecture with respect to the AUC. With respect to the convolutional layers, we evaluated different numbers of neuronal units (16 to 2048 in powers of two) and the number of filters (16 to 2048 in powers of two) for each particular convolutional layer. We further applied different kernel sizes (2x2 to 5x5) and evaluated different configurations of the max pooling layers (2x2 to 4x4). As activation functions we used ReLUs and sigmoid. Dropout layers were evaluated using dropout rates from 0.1 to 0.9 in steps of 0.1. Further, we added batch normalization layers to evaluate their benefits with respect to overﬁtting and convergence. We used binary cross entropy as loss function together with the Adam or the RMSprop optimizer. We evaluated different learning rates (0.0001, 0.0002, 0.001, 0.002, and 0.01) and used batch sizes from one to 128 in powers of two. The final model was trained with an Adam optimizer with a learning rate of 0.0002, a batch size of 32 and for 100 epochs. Training was performed on a LINUX machine using Ubuntu 16.04 LTS and a Nvidia GTX 1080 TI graphics card.

Figure S1. Model architecture. The model consists of a series of chained layers (convolutional layers (white), max-pooling layers (red) and fully connected layer (green)). The numbers indicate the width, height and depth of each particular layer. Information is passed from the raw image data (gray layer) forward through the network. By stacking convolutional layers and applying a pooling operation, the extracted features become larger and more complex. The last few network layers cast the feature-filtered image data to votes (PBL being prevalent or not).

***Modelling via CNNs***

**Table S2.** Example for a group shuffling schema.

| **Fold number** | **Patients in the validation sets** |
| --- | --- |
| 1 | 0 19 20 28 30 31 32 48 52 53 57 60 61 63 69 70 77 |
| 2 | 2 3 4 6 7 14 18 26 36 39 42 45 52 54 71 80 81 |
| 3 | 4 6 9 17 20 32 36 38 49 50 55 71 73 74 77 78 81 |
| 4 | 6 14 33 35 37 39 45 52 55 58 59 60 62 63 66 73 76 |
| 5 | 5 6 9 13 15 20 38 40 42 53 57 59 60 61 64 67 68 |
| 6 | 1 4 15 22 24 25 34 37 41 45 46 52 75 78 79 80 84 |
| 7 | 2 8 14 23 34 36 38 44 47 48 54 59 61 64 73 74 85 |
| 8 | 3 4 18 22 33 39 40 41 42 51 59 61 64 70 75 79 84 |
| 9 | 2 5 6 13 15 16 17 20 27 31 33 43 44 54 64 71 82 |
| 10 | 4 6 8 13 16 22 24 27 30 33 35 40 52 67 69 74 84 |

***Image Augmentation***

We applied several image augmentation techniques such as feature-wise center, zca whitening, rescaling, shearing, zooming, rotating and ﬂipping images horizontally and vertically (Hussain et al. 2017). We found image rotation (± 20 degrees), shearing (from 0.8 to 1.2) and zooming (from 0.8 to 1.2) as most appropriate.

***Examples of classified images***

Top 10 true positive (TP), true negative (TN), false positive (FP) and false negative for the base line model, per validation fold

# True Positive (TP)

# Validation fold 1


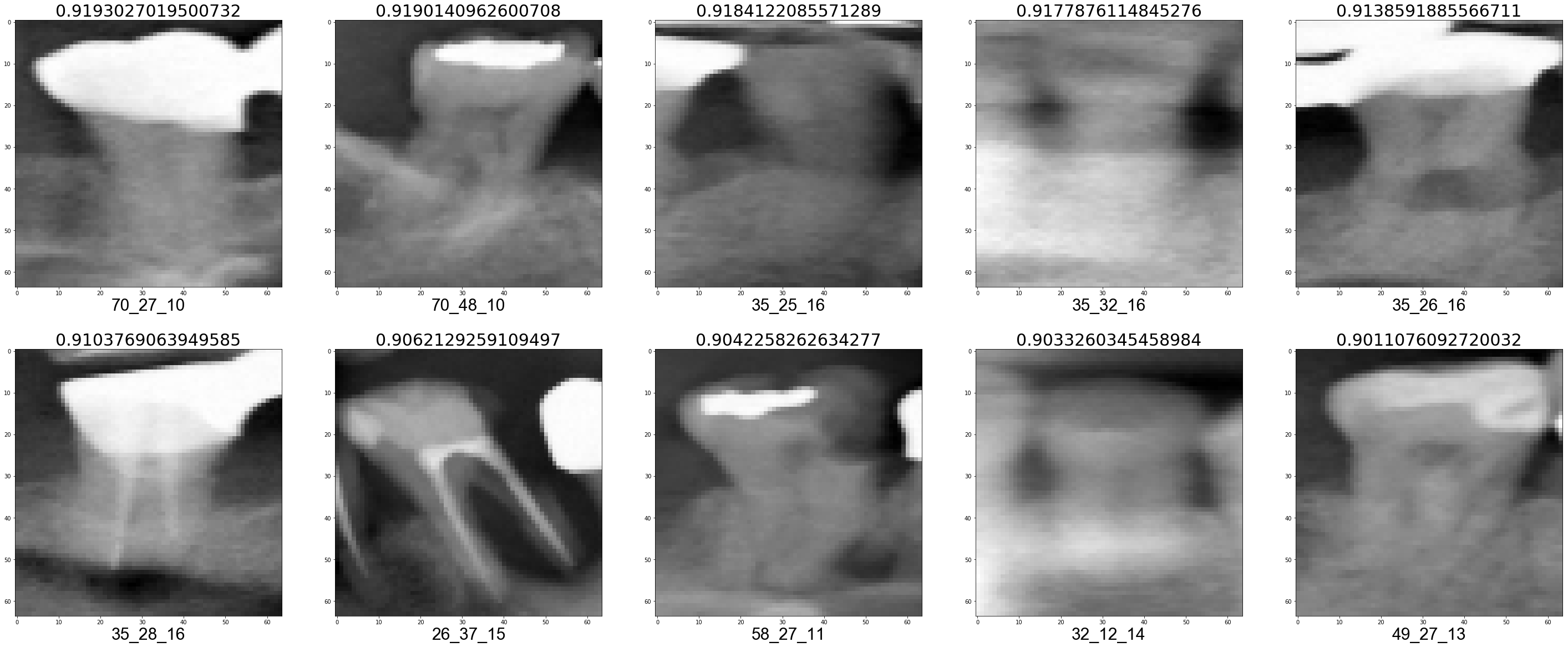


# Validation fold 2

#
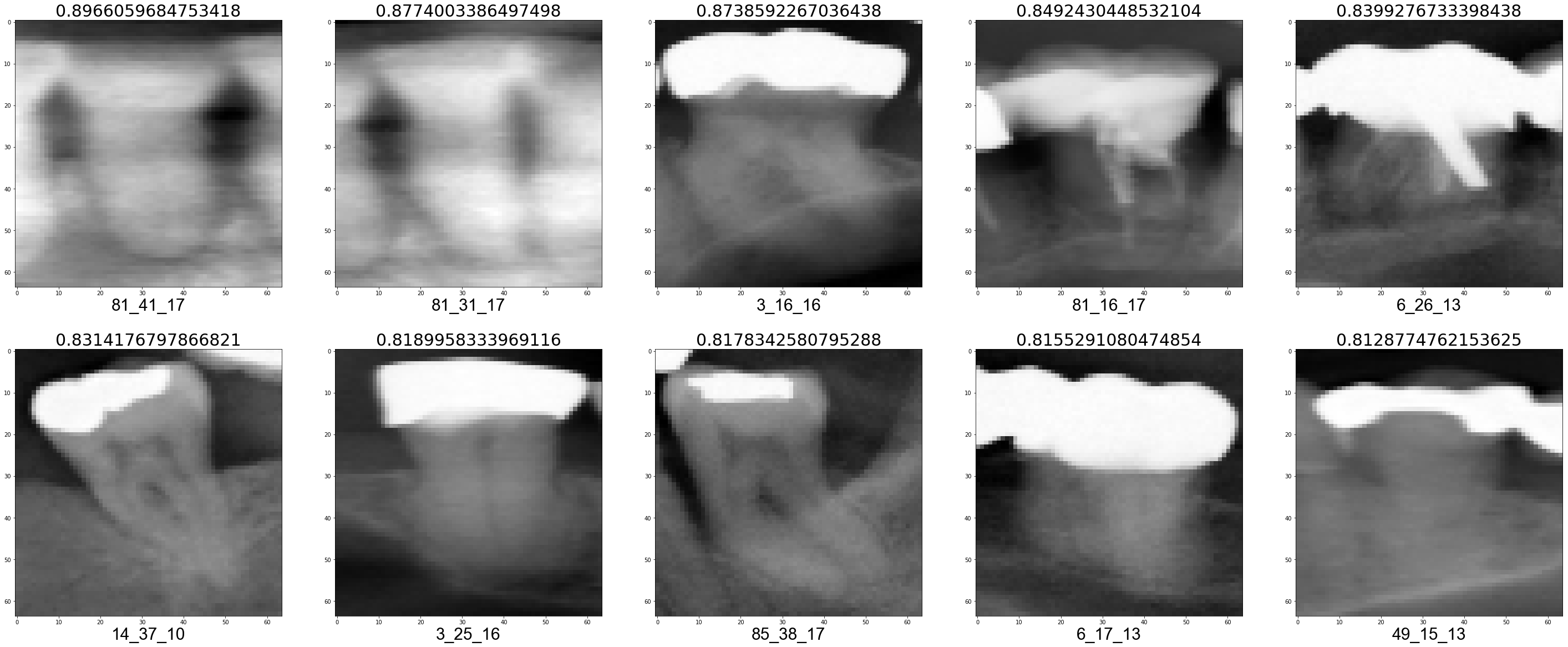


# Validation fold 3

#
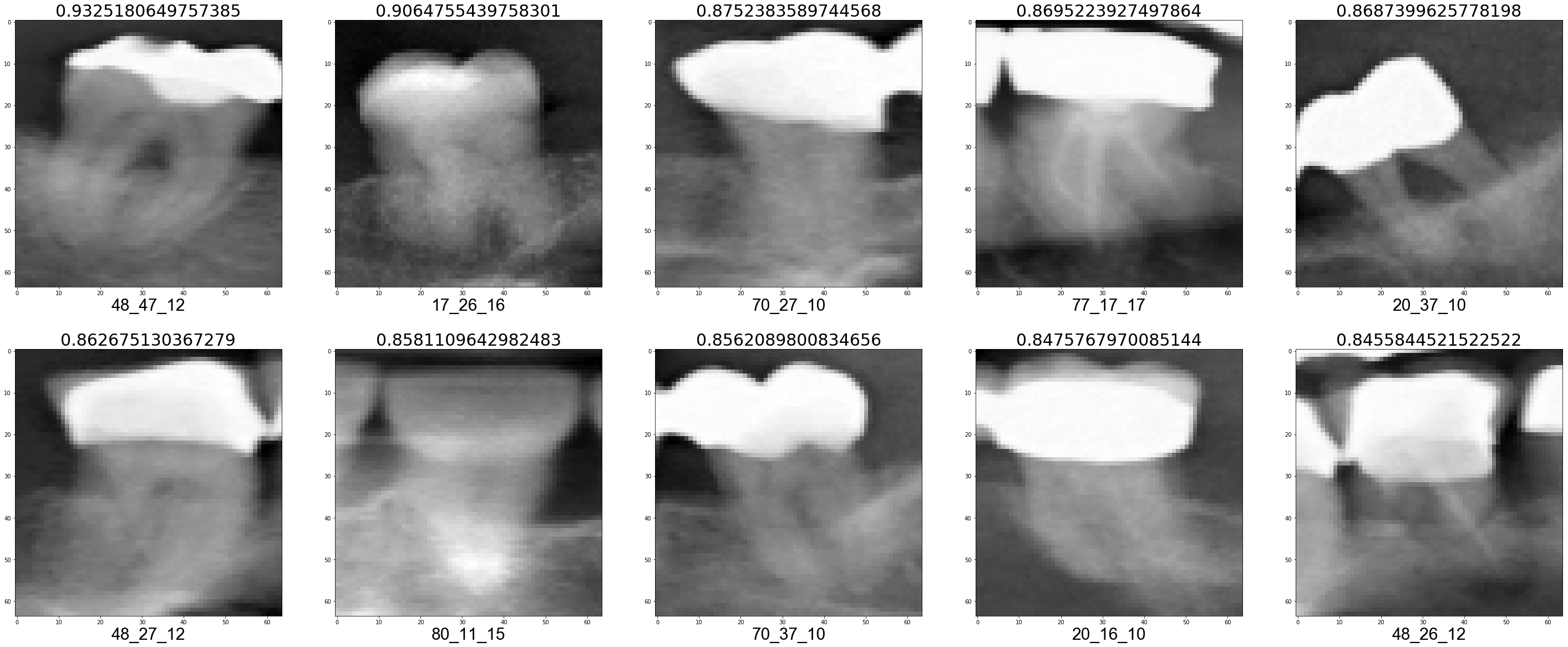


# Validation fold 4

#
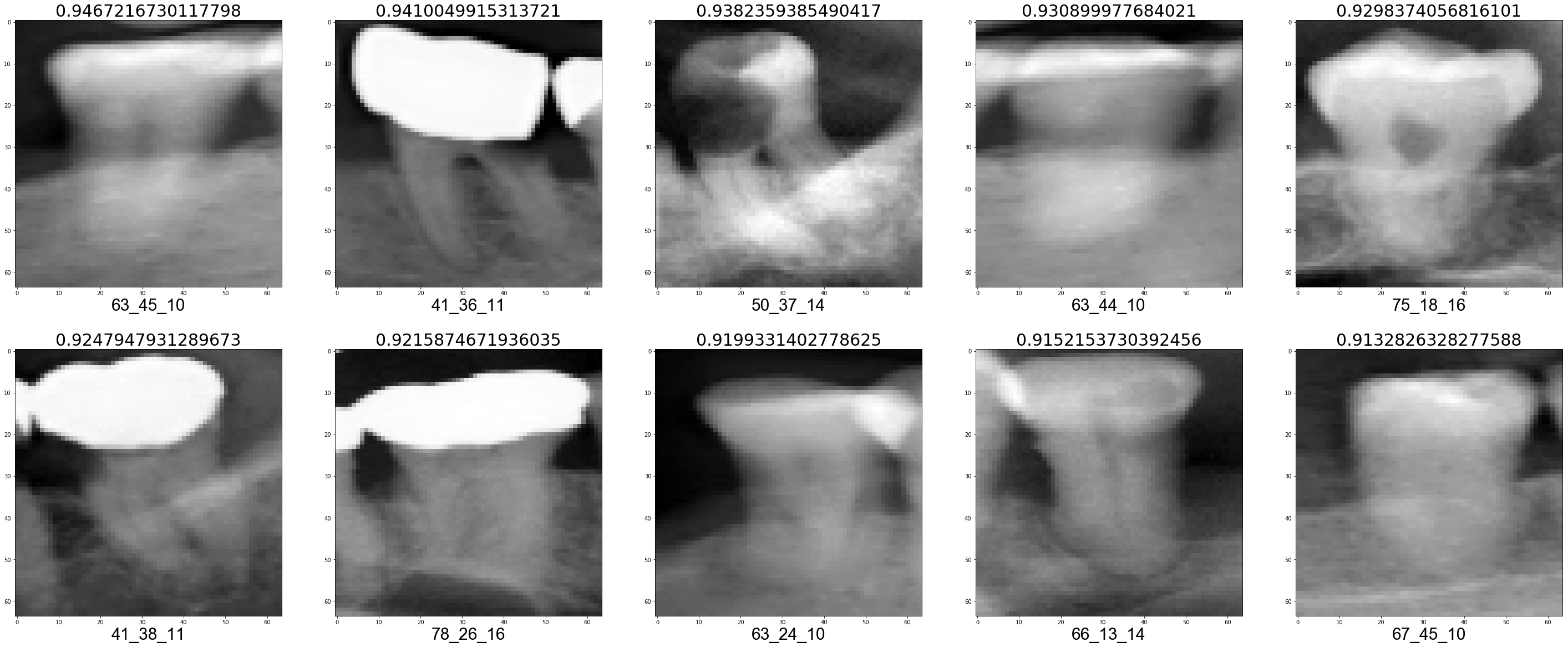


# Validation fold 5

#
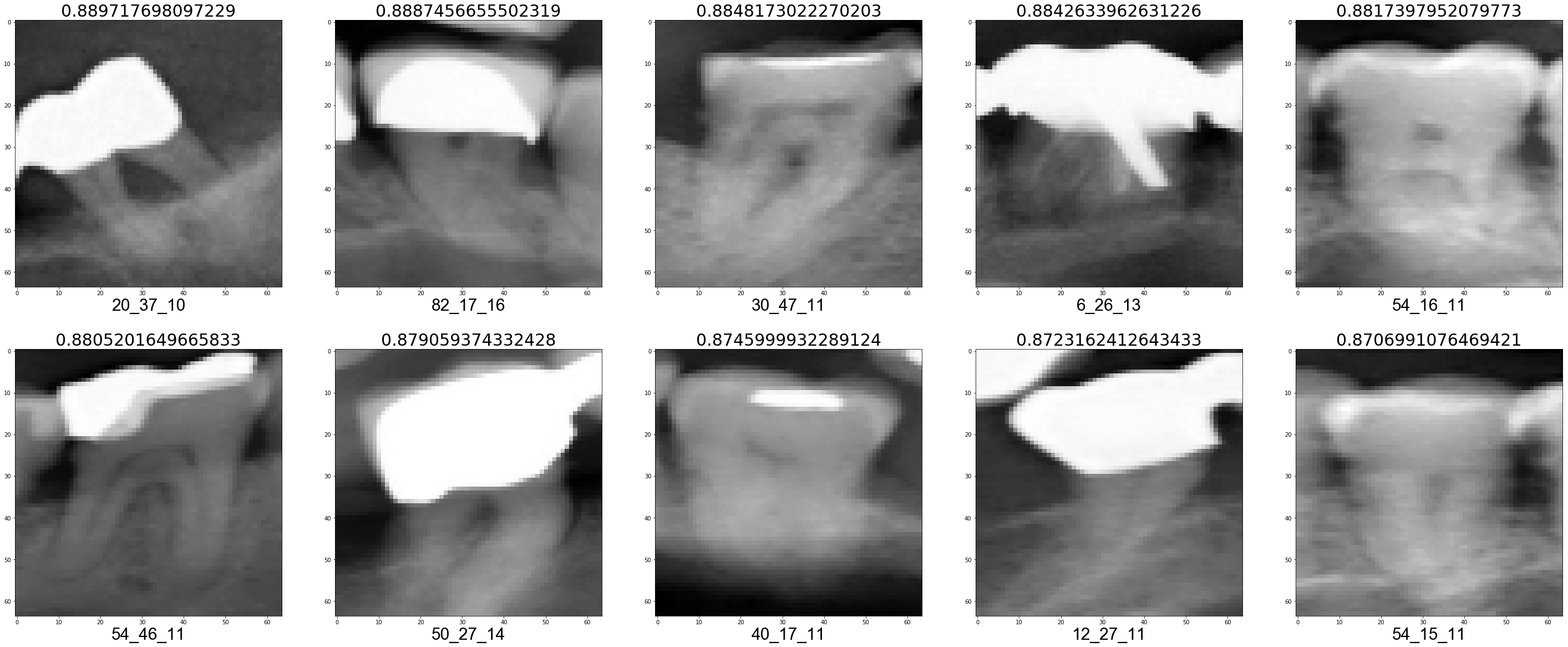


# Validation fold 6

#
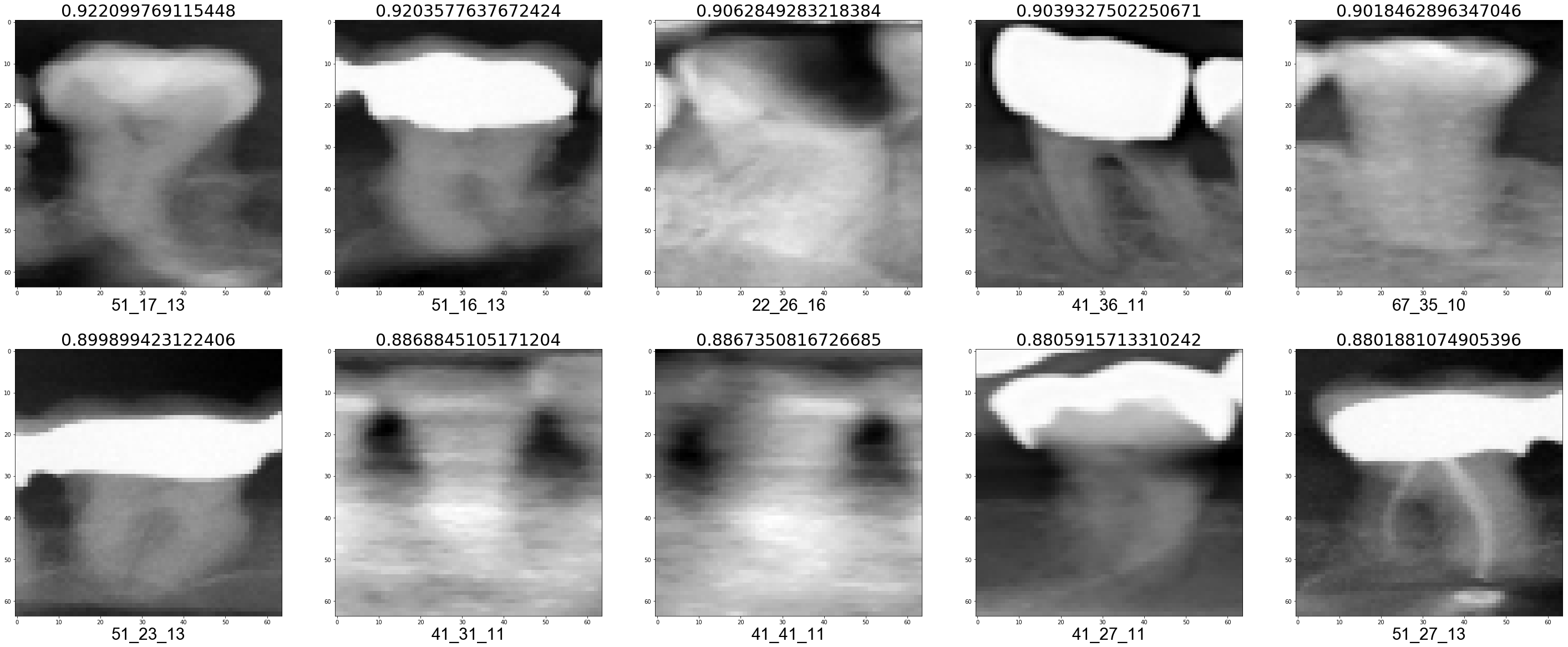


# Validation fold 7

#
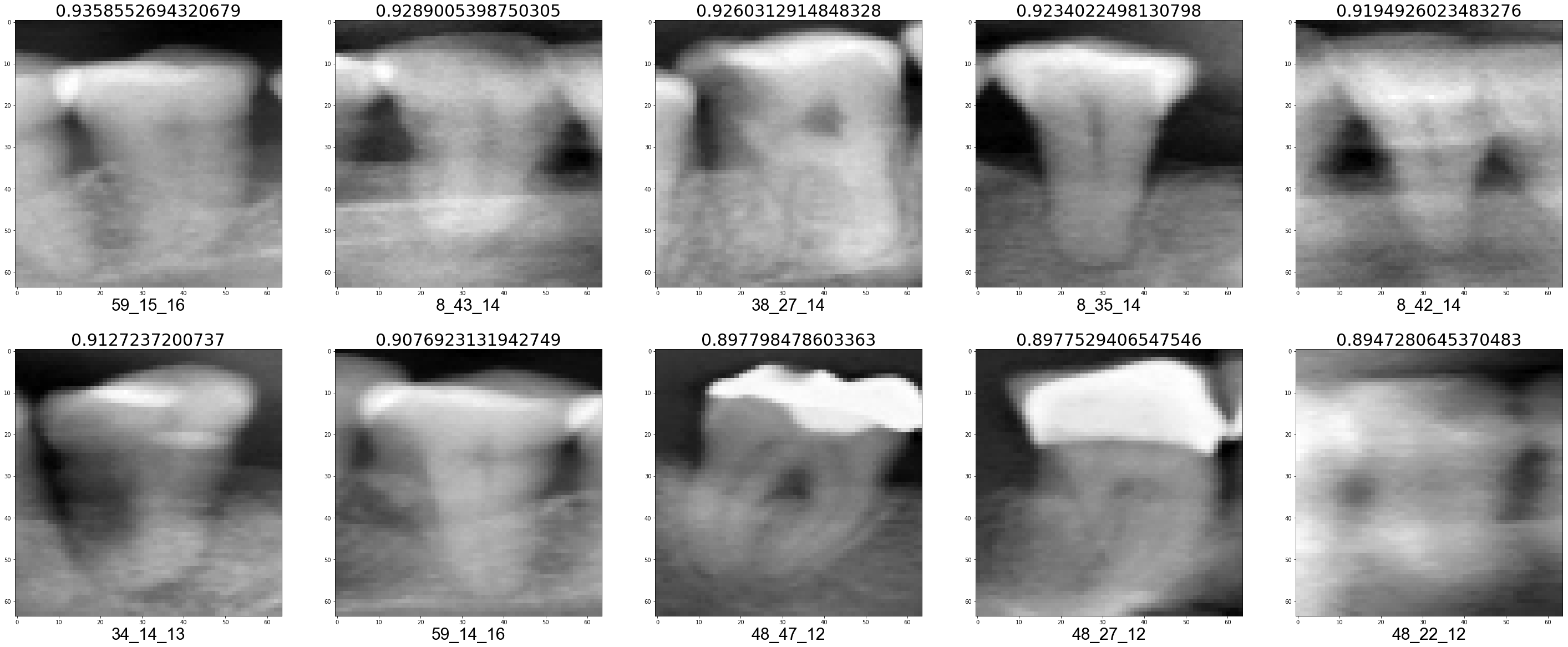


# Validation fold 8

#
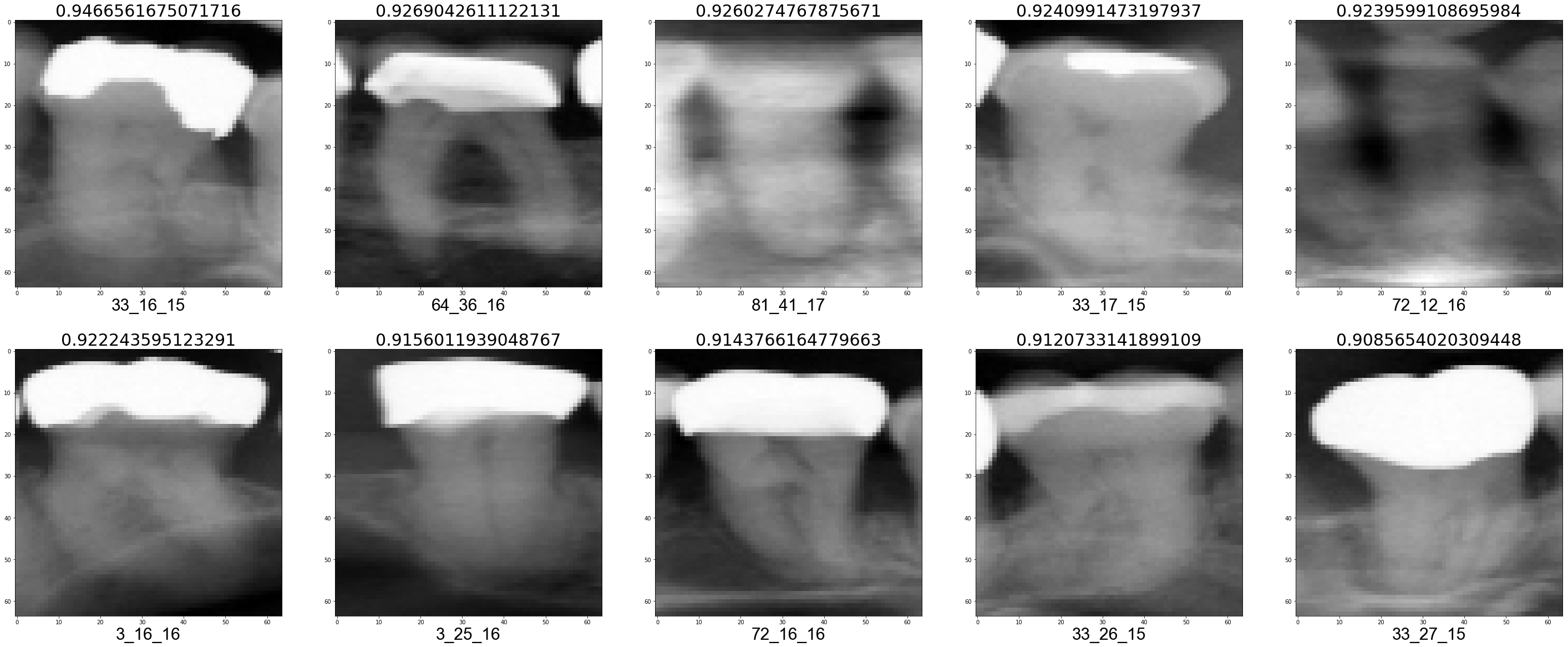


# Validation fold 9

#
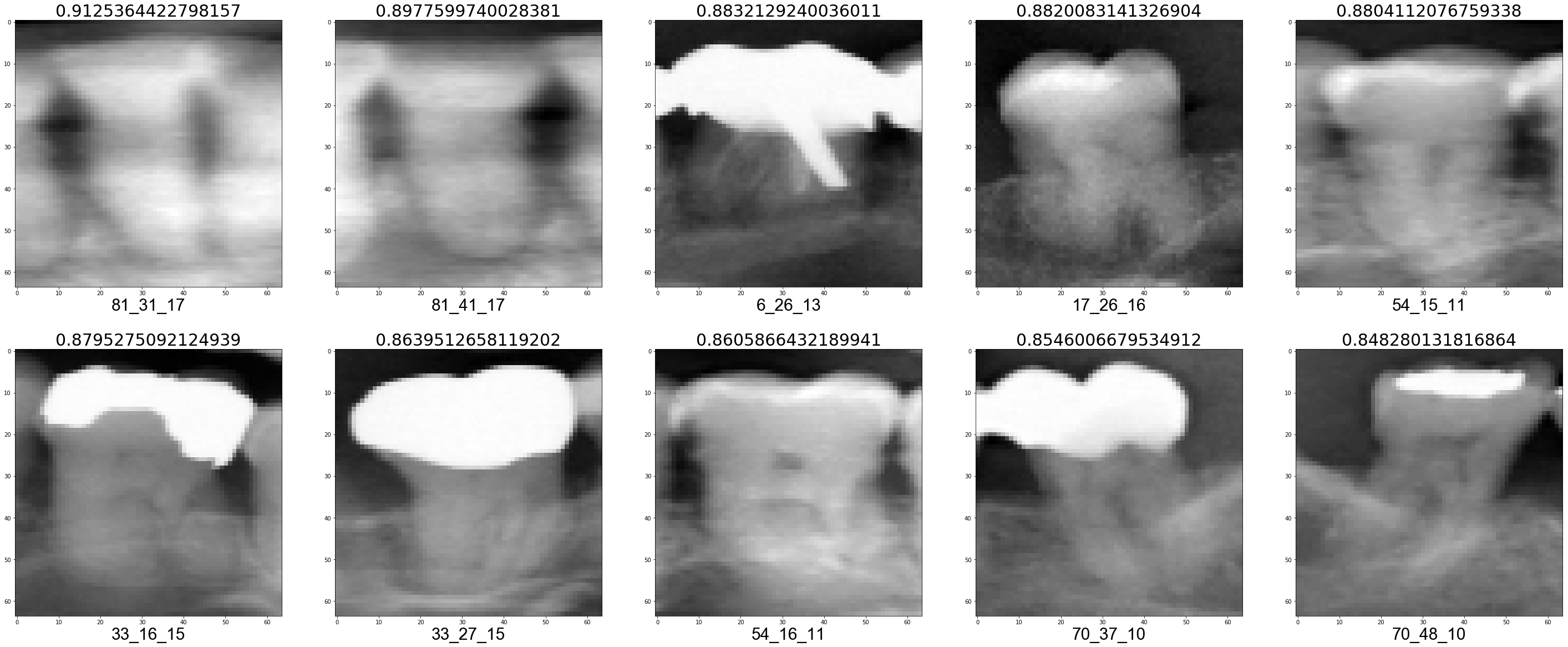


# Validation fold 10

#
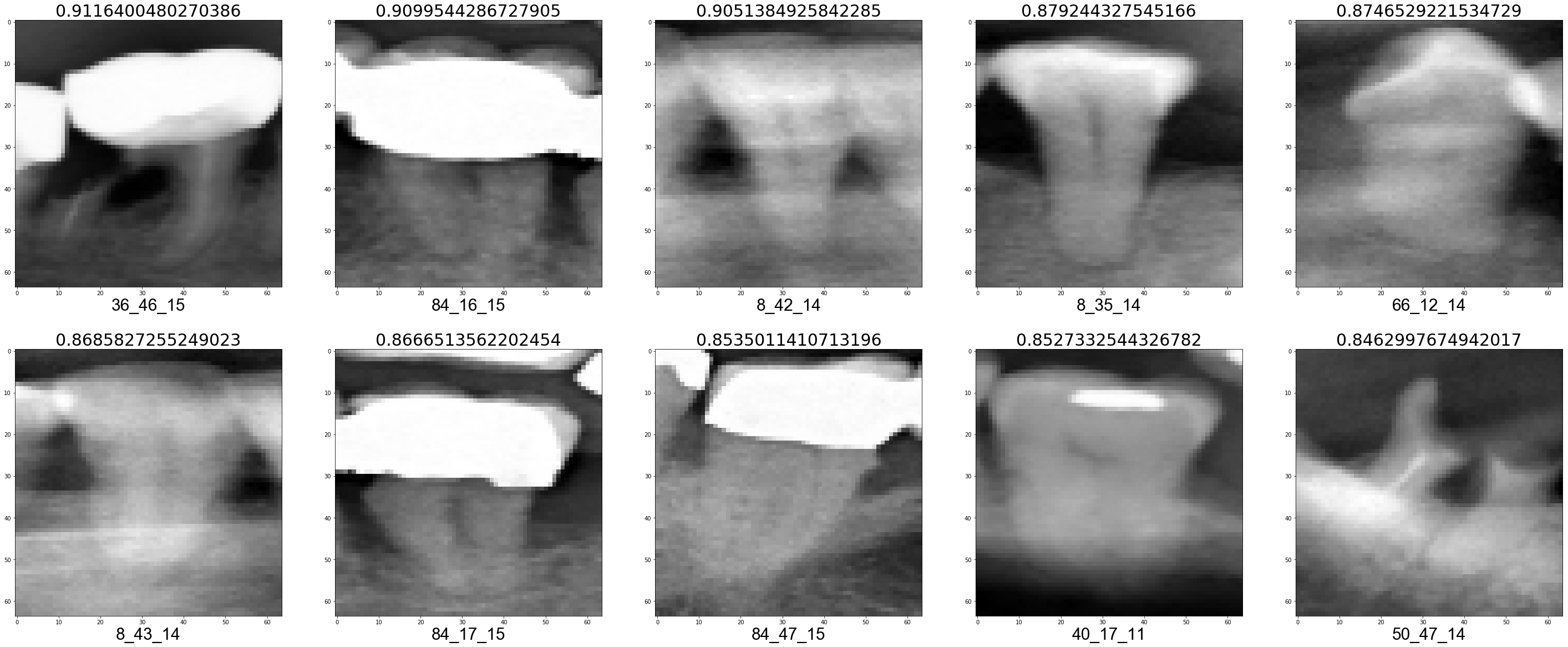


# True Negative (TN)

# Validation fold 1

#
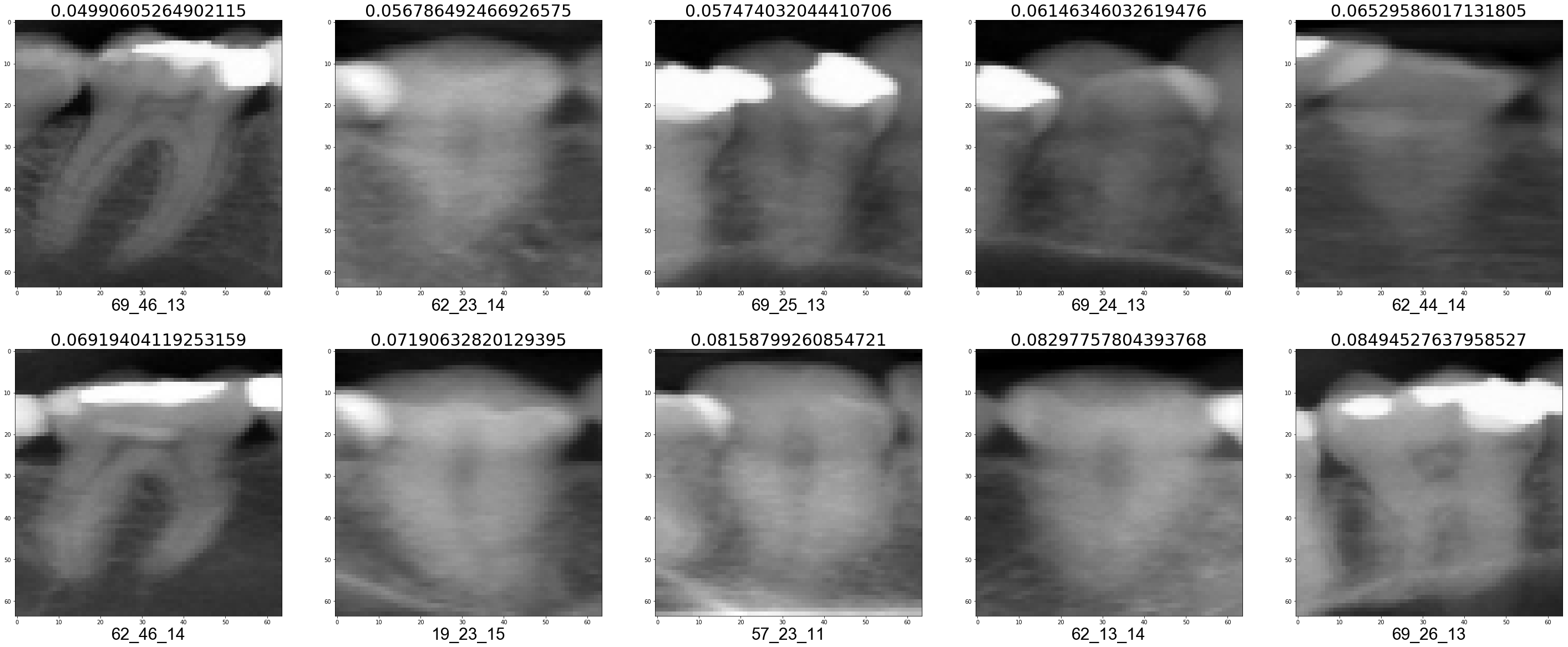


# Validation fold 2

#
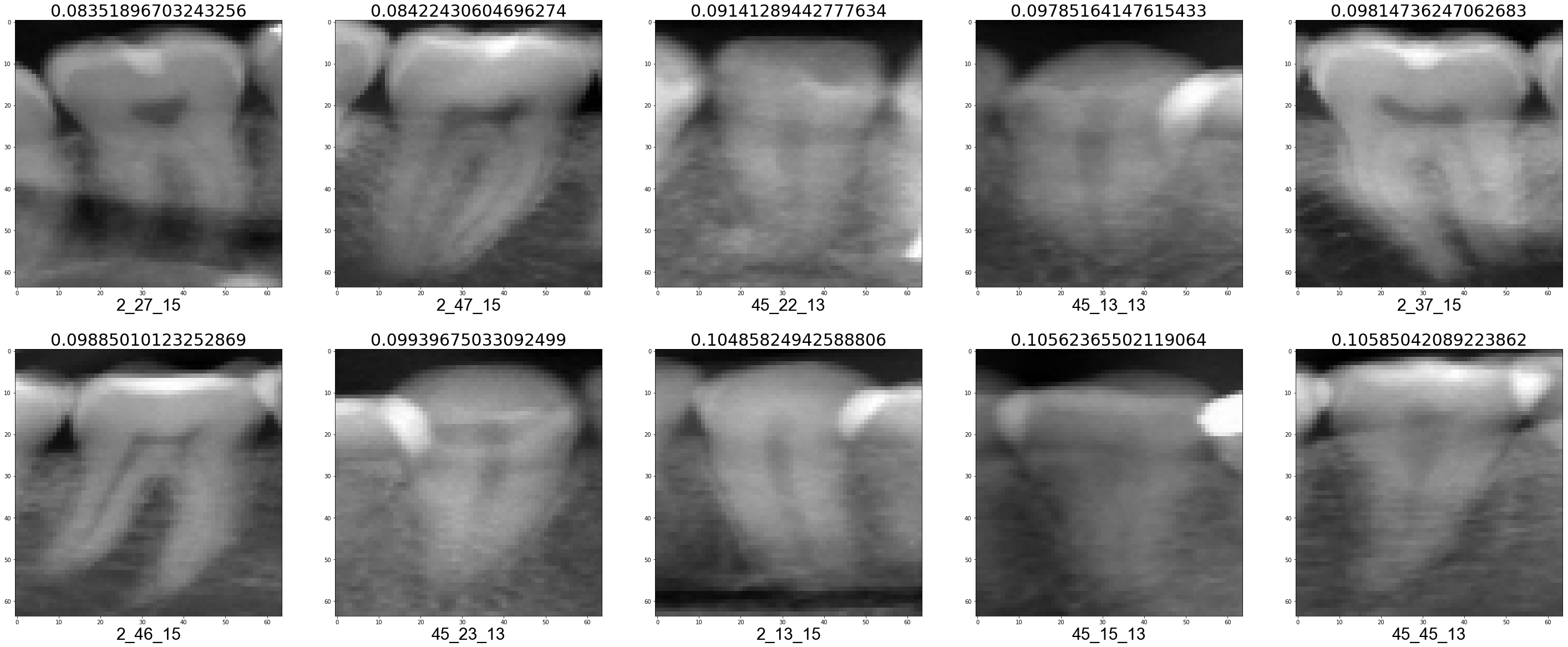


# Validation fold 3

#
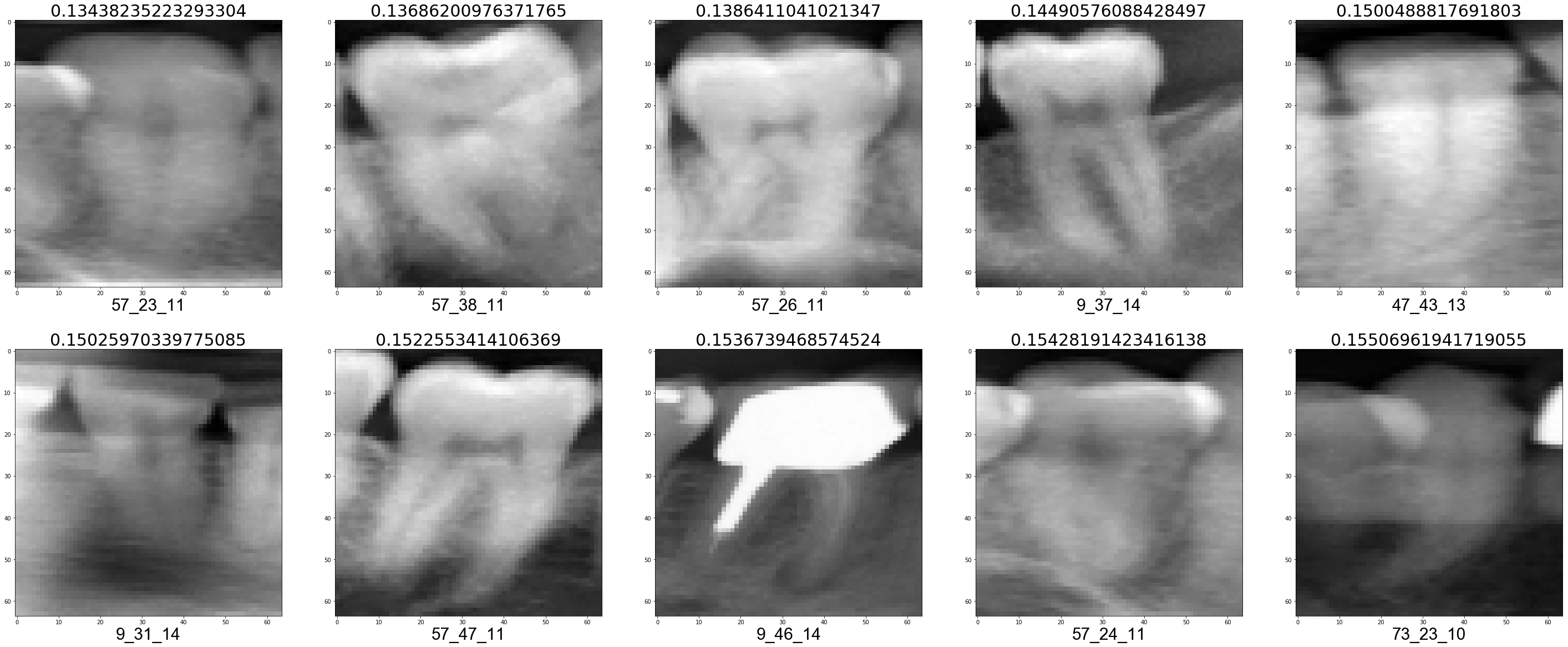


# Validation fold 4

#
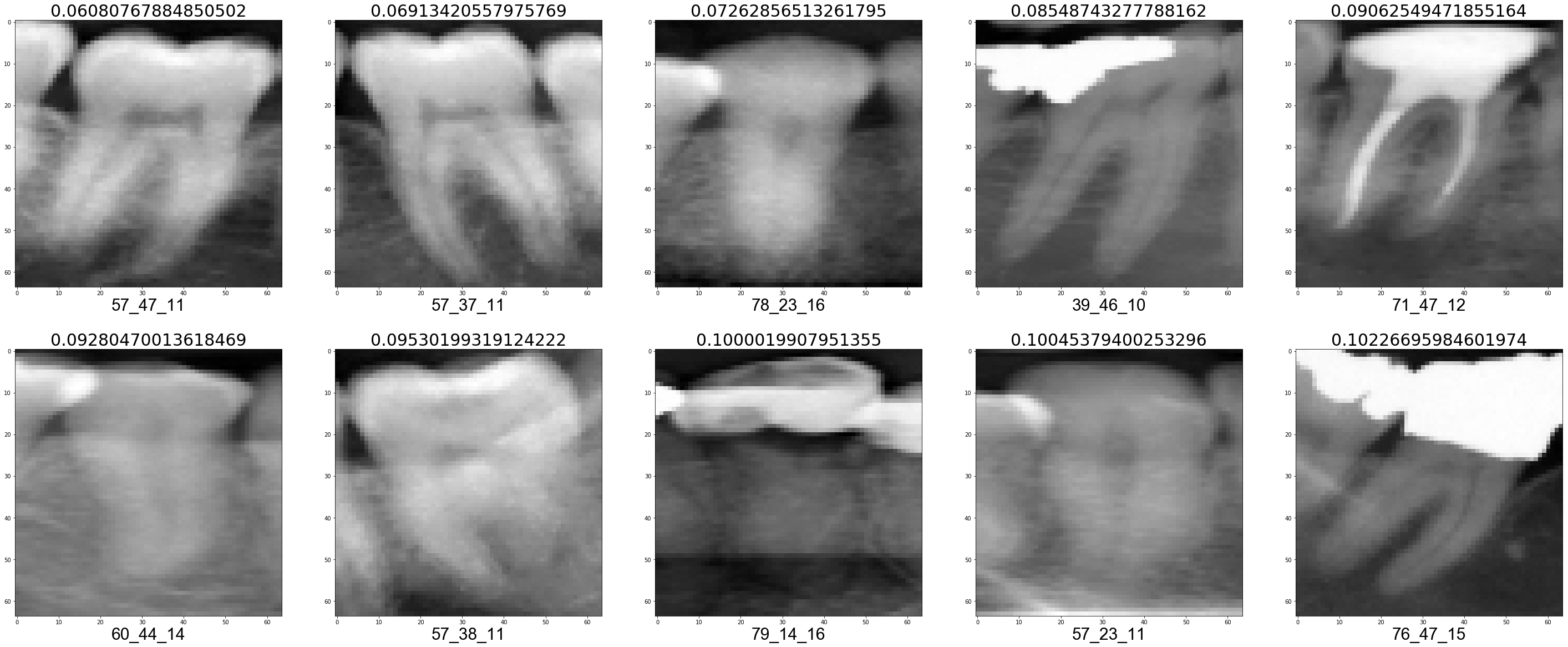


# Validation fold 5

#
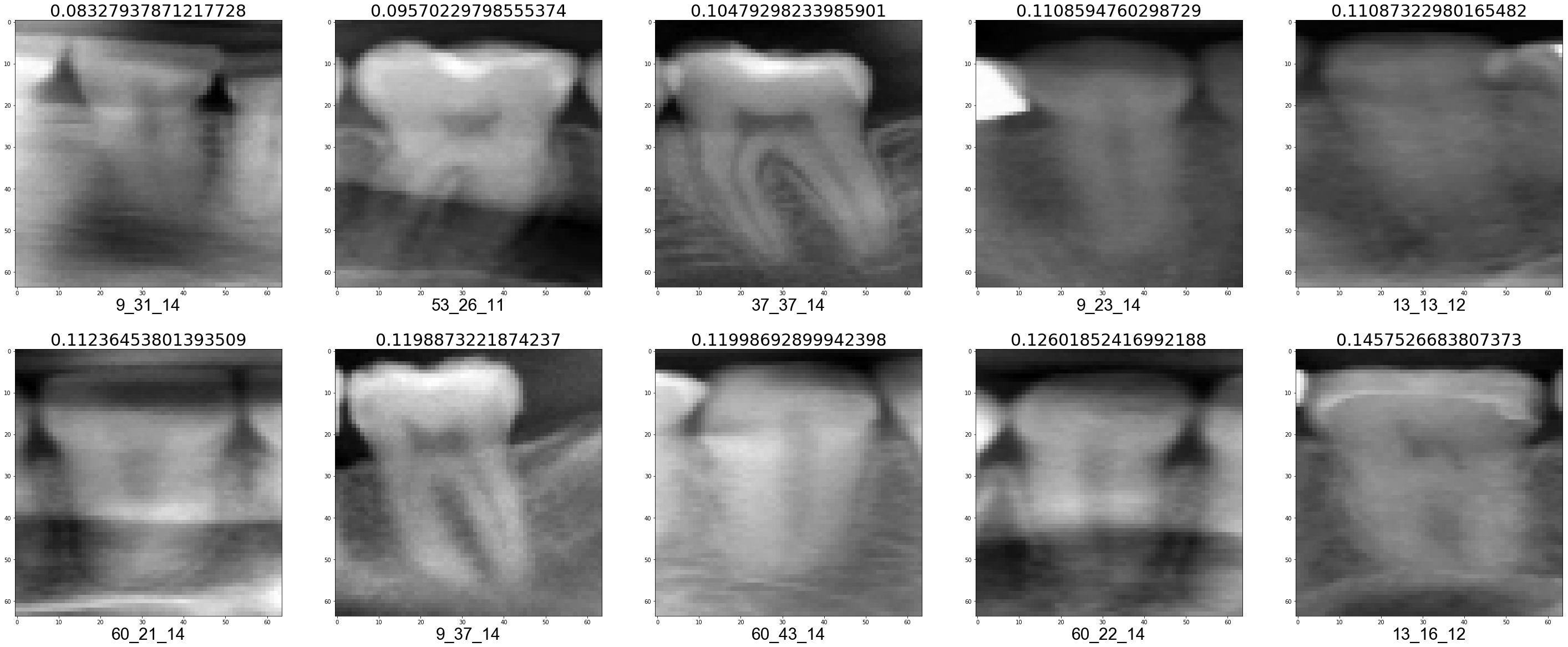


# Validation fold 6

#
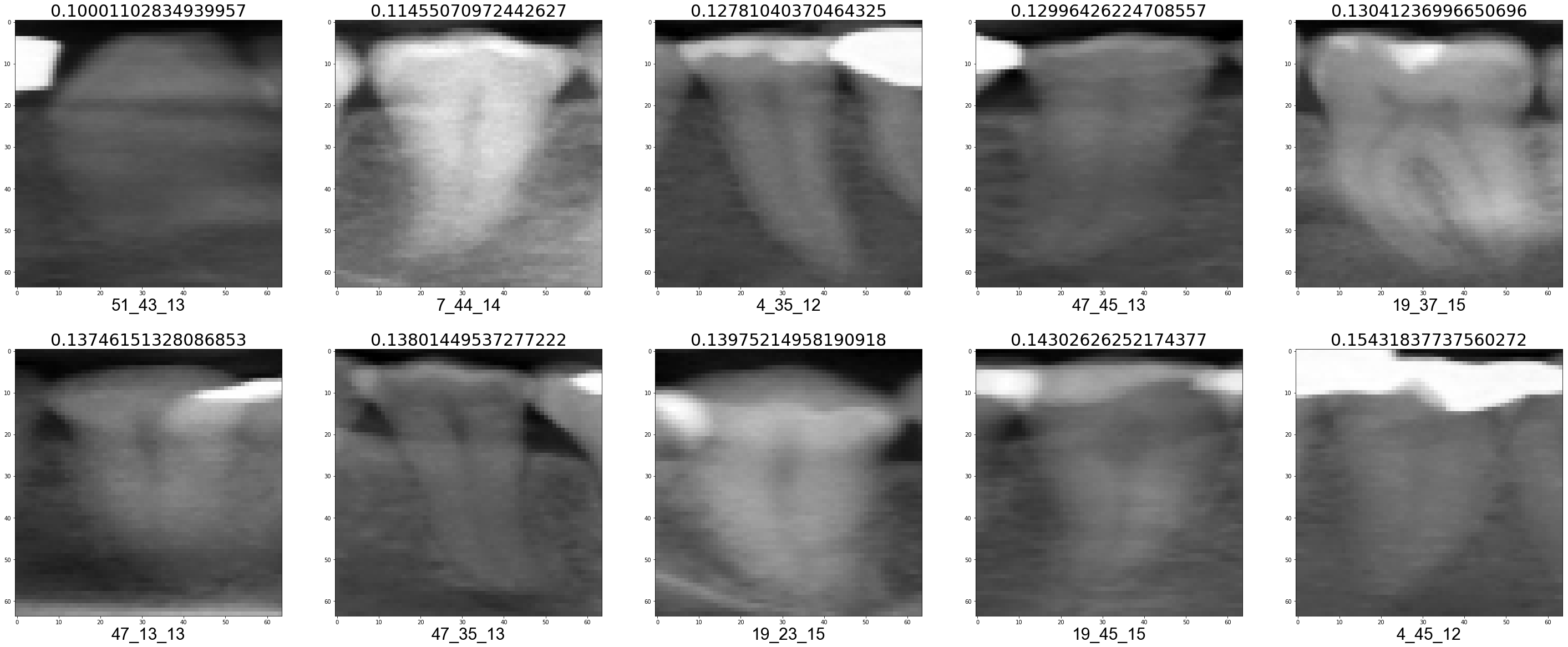


# Validation fold 7

#
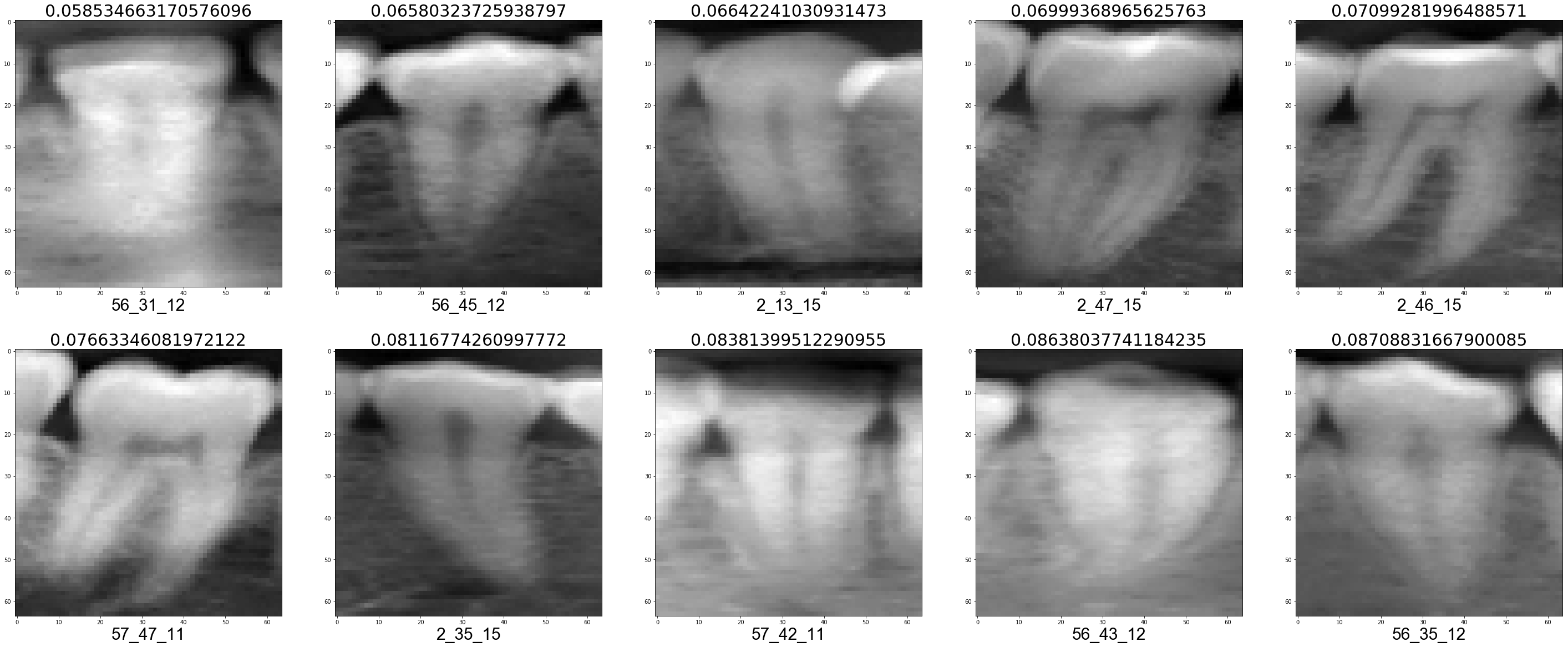


# Validation fold 8

#
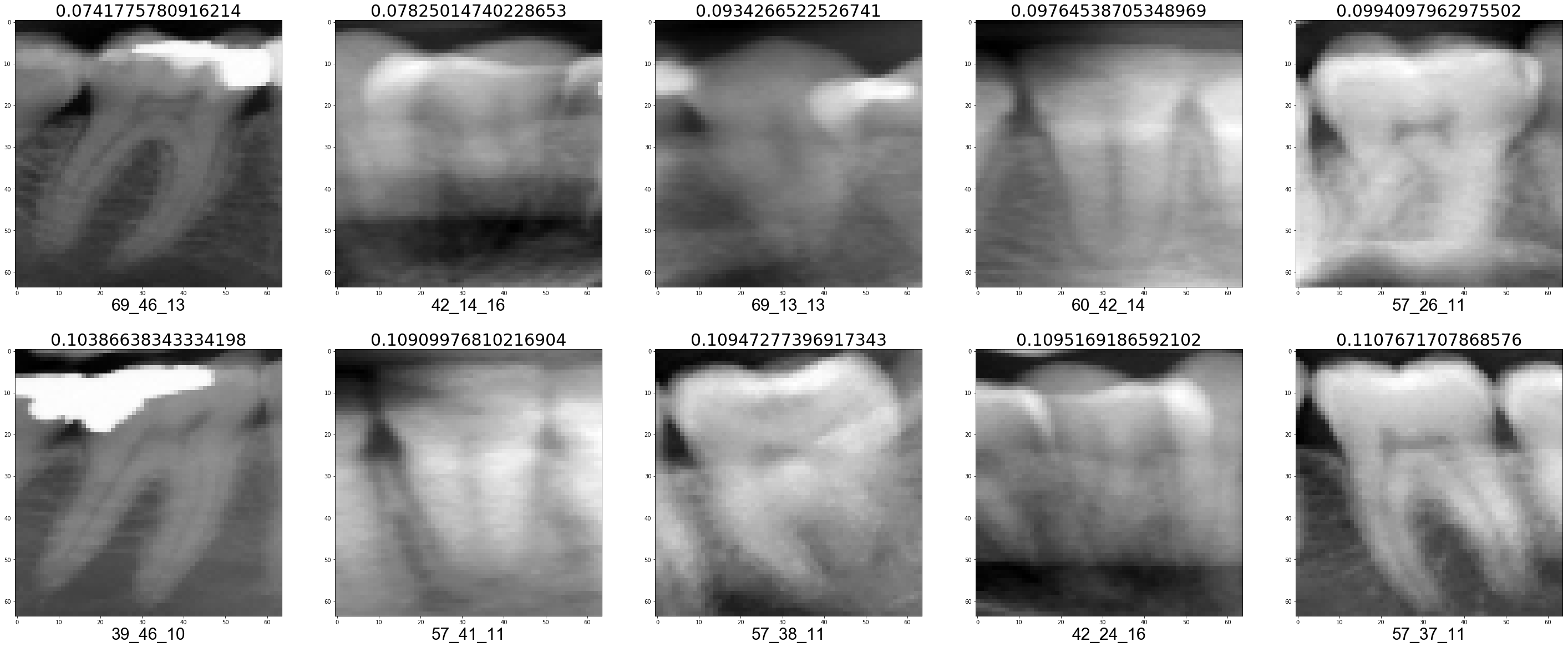


# Validation fold 9

#
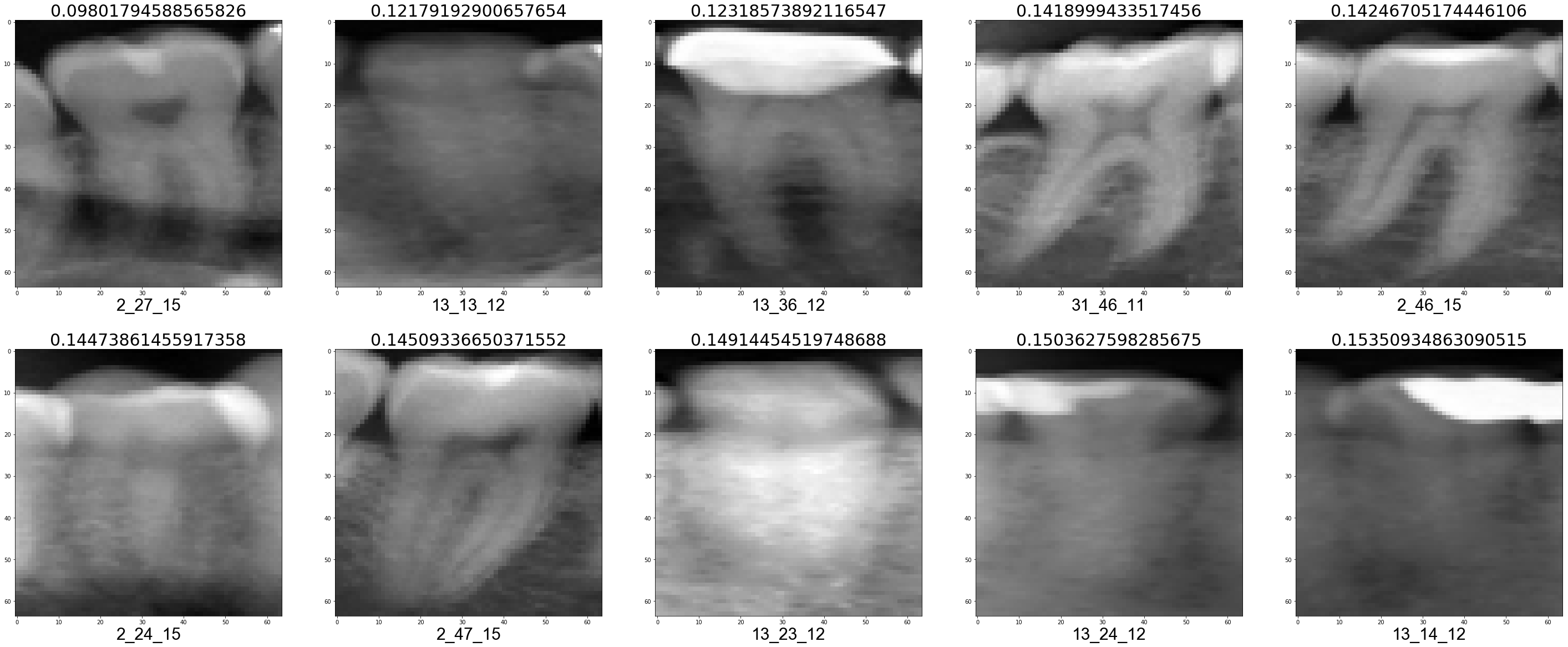


# Validation fold 10
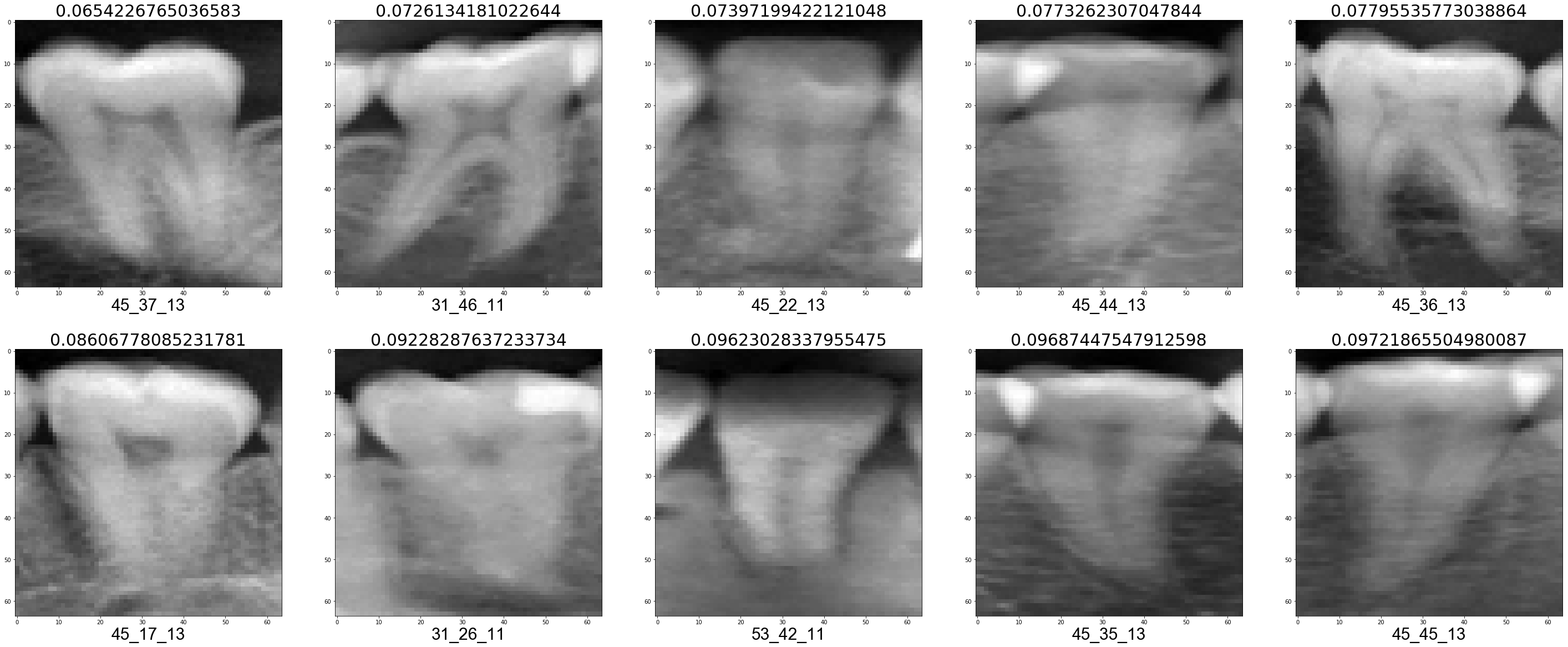


# False Positive (FP)

# Validation fold 1

#
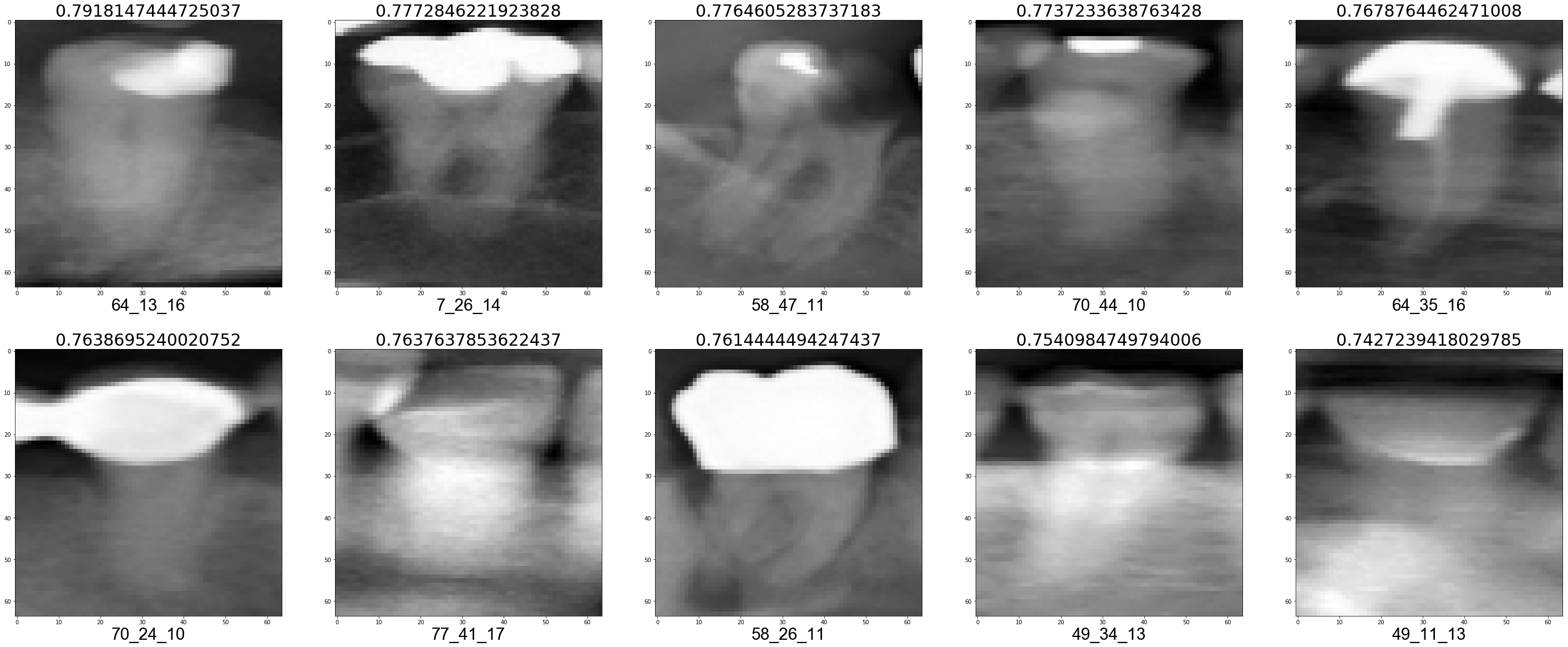


# Validation fold 2

#
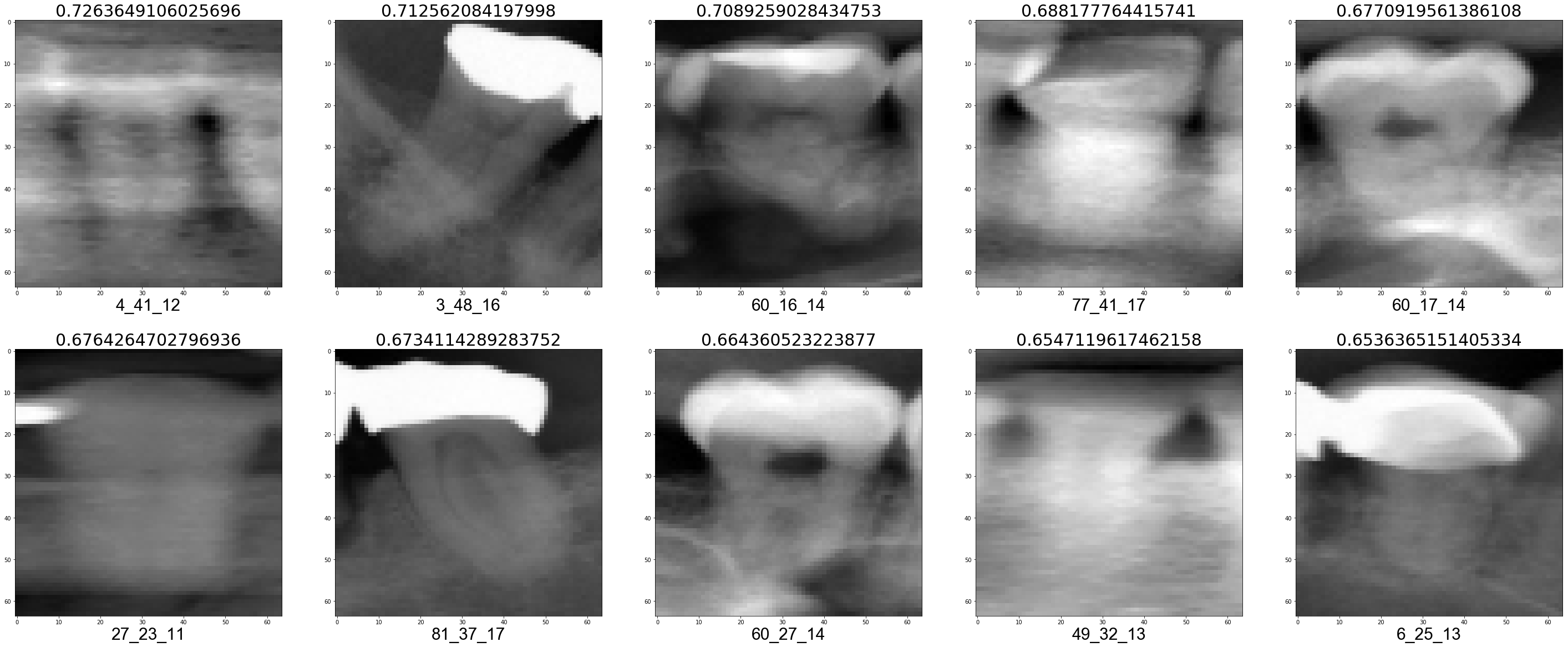


# Validation fold 3

#
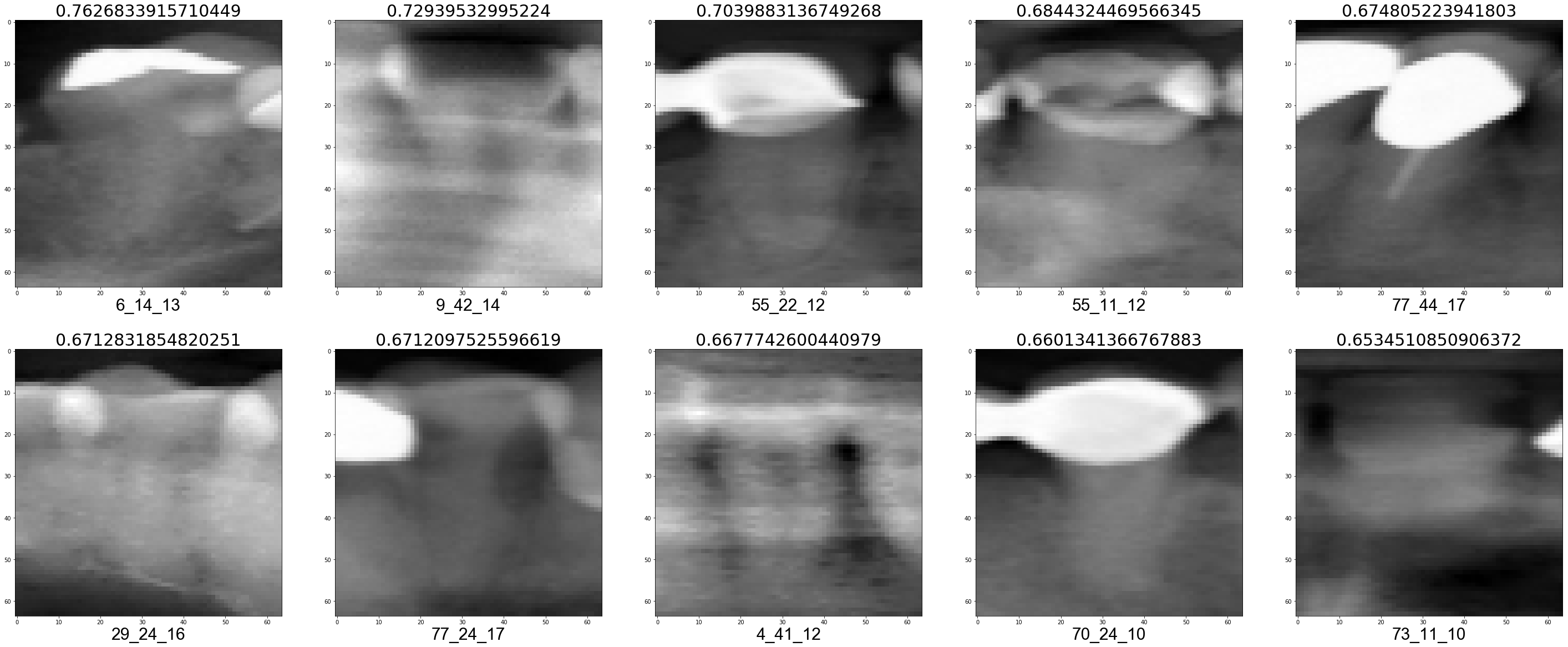


# Validation fold 4

#
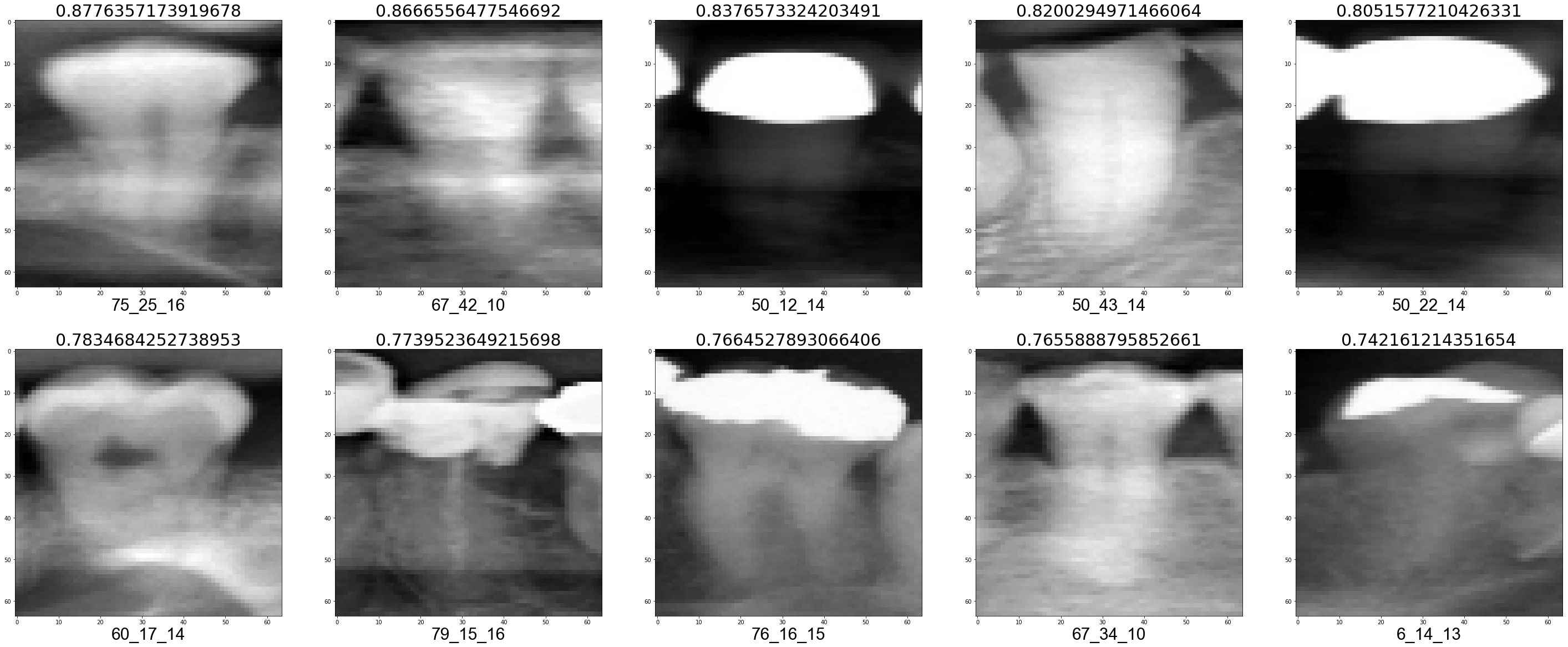


# Validation fold 5

#
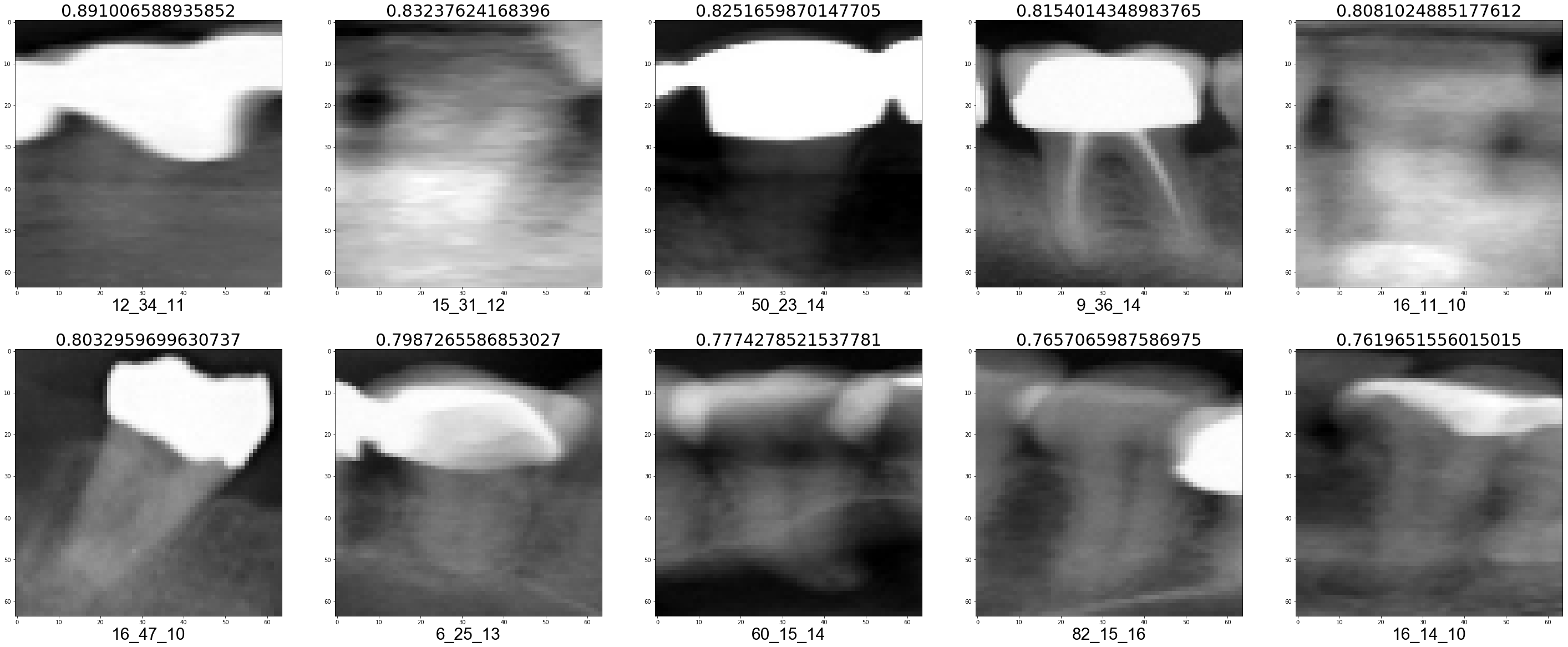


# Validation fold 6

#
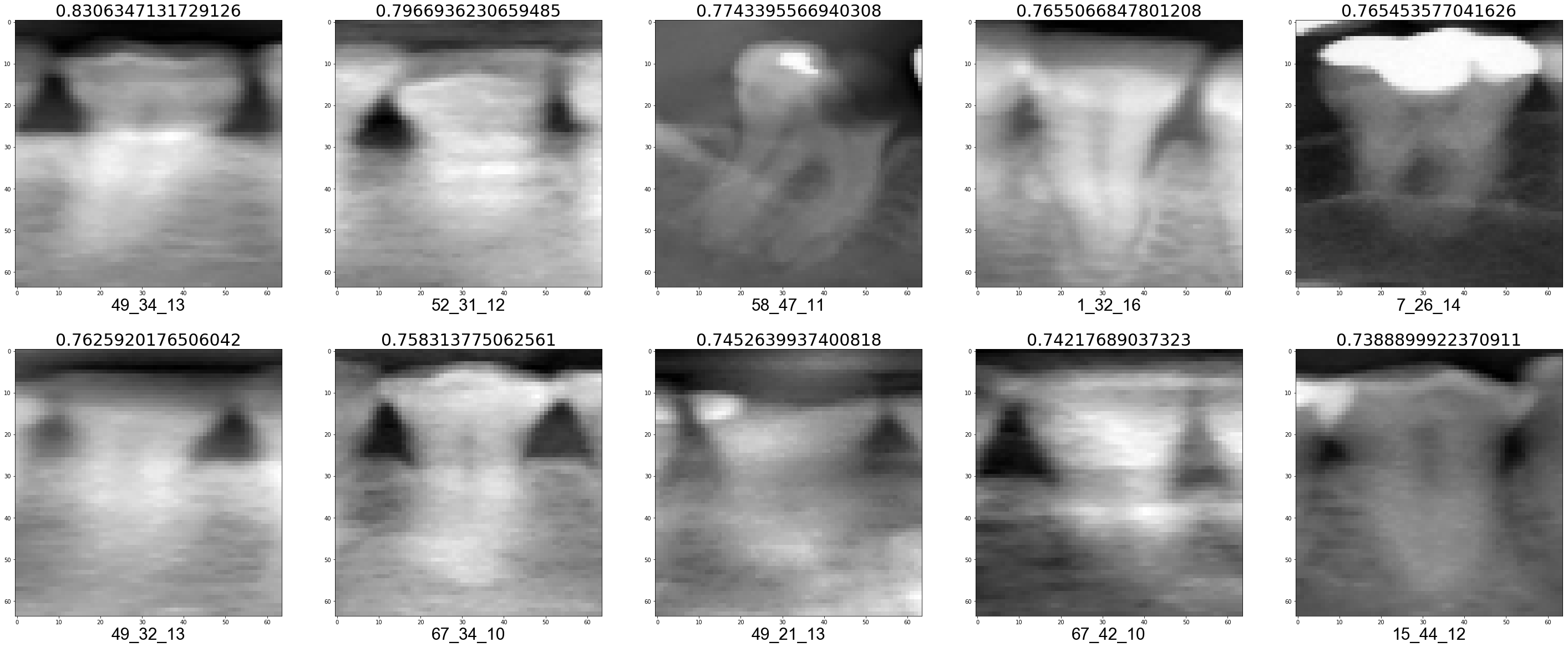


# Validation fold 7

#
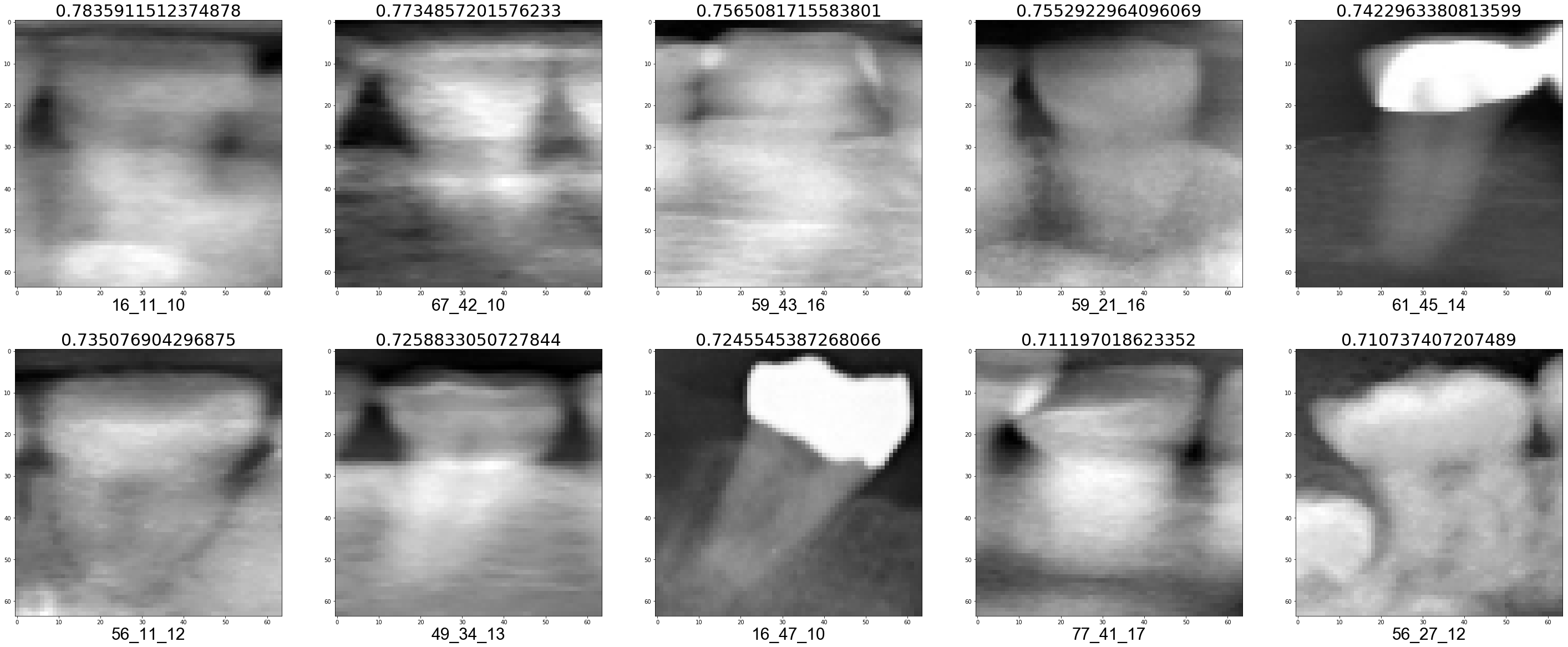


# Validation fold 8

#
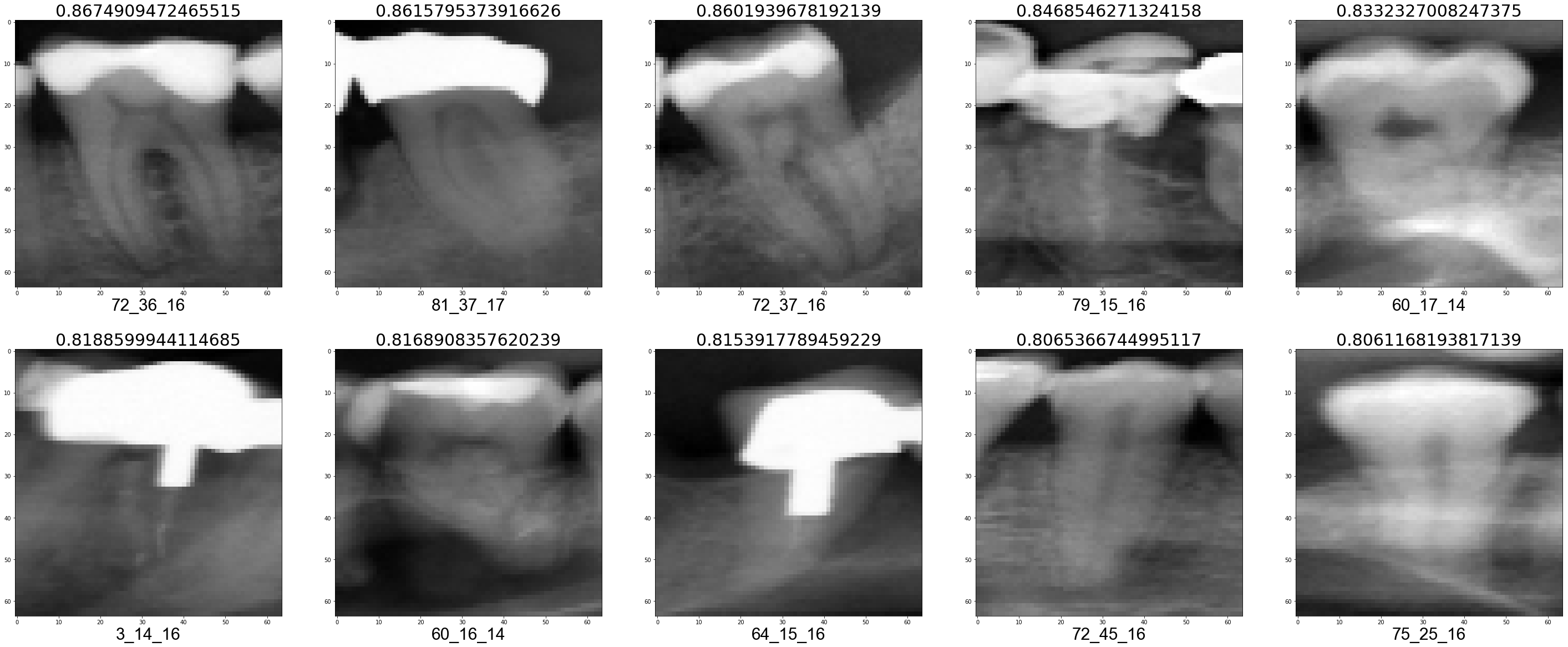


# Validation fold 9

#
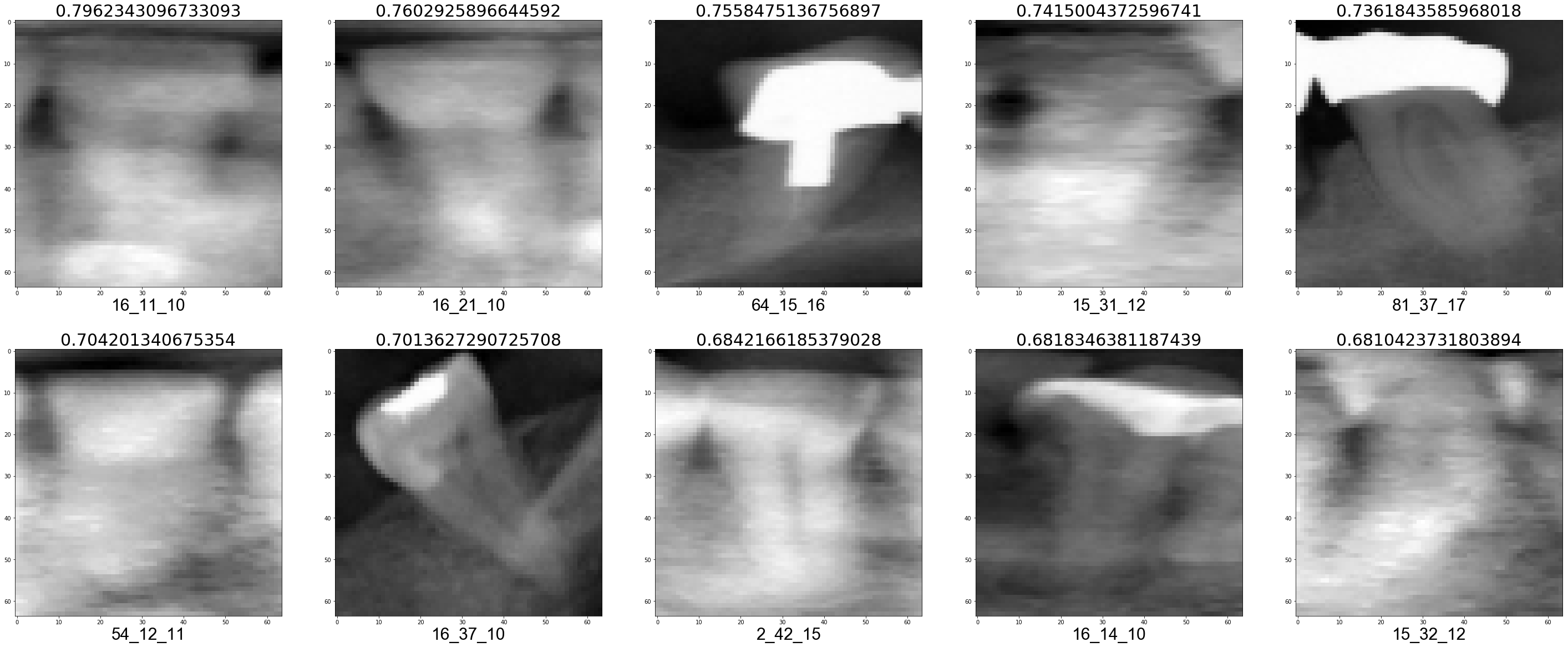


# Validation fold 10

#
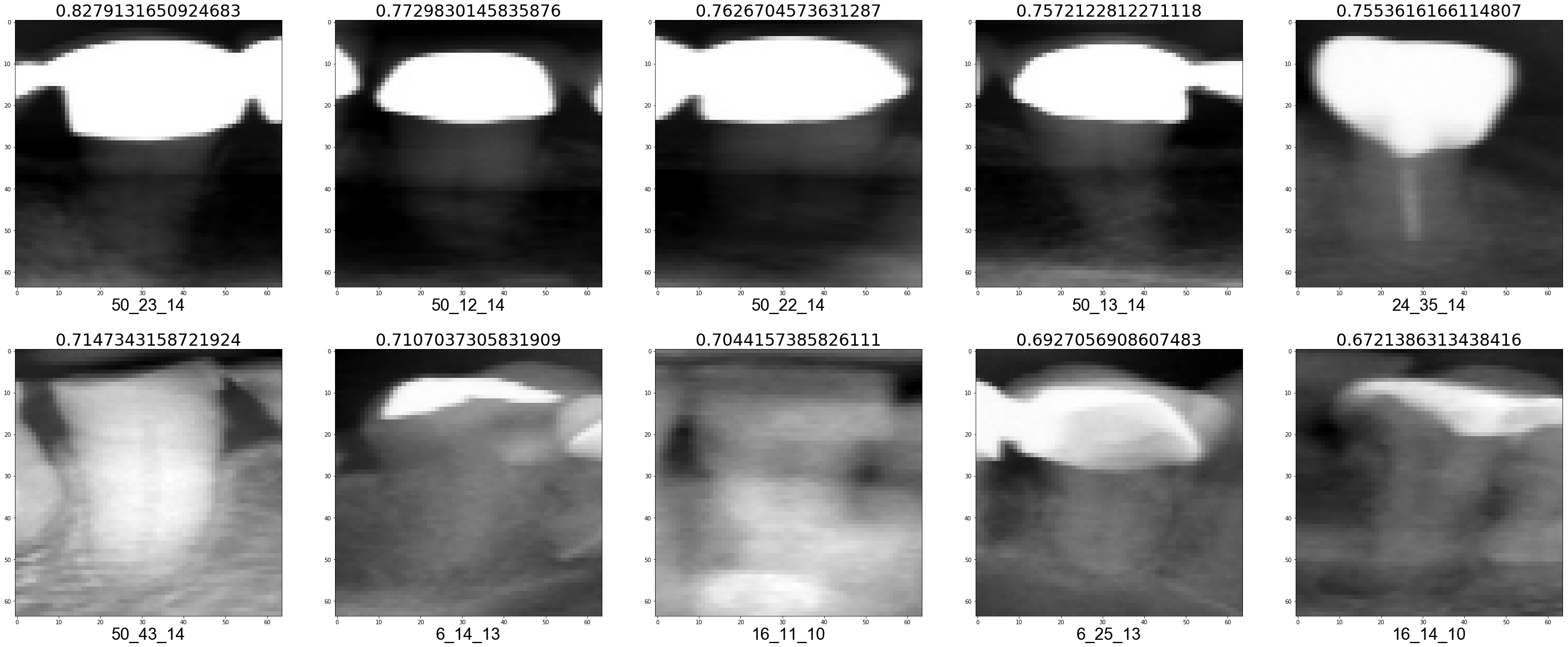


# False Negative (FN)

# Validation fold 1

#
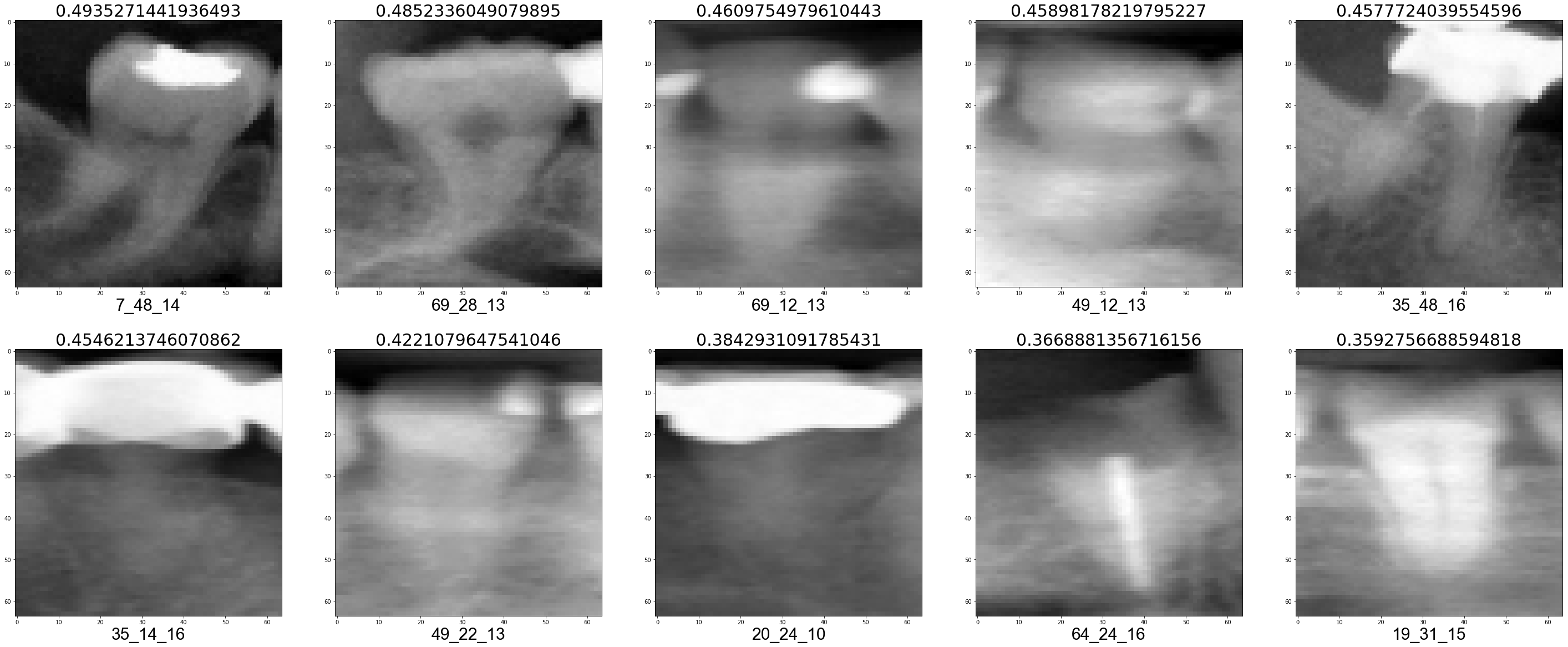


# Validation fold 2

#
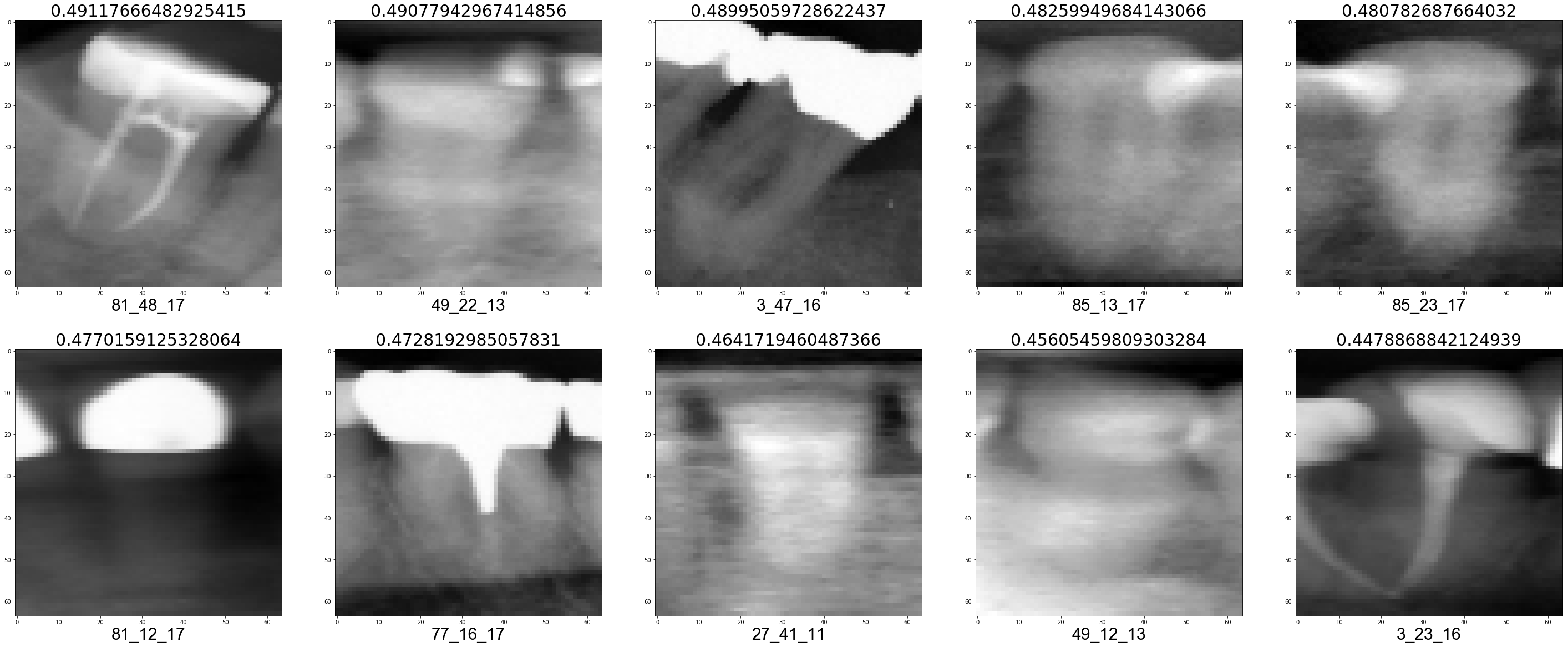


# Validation fold 3

#
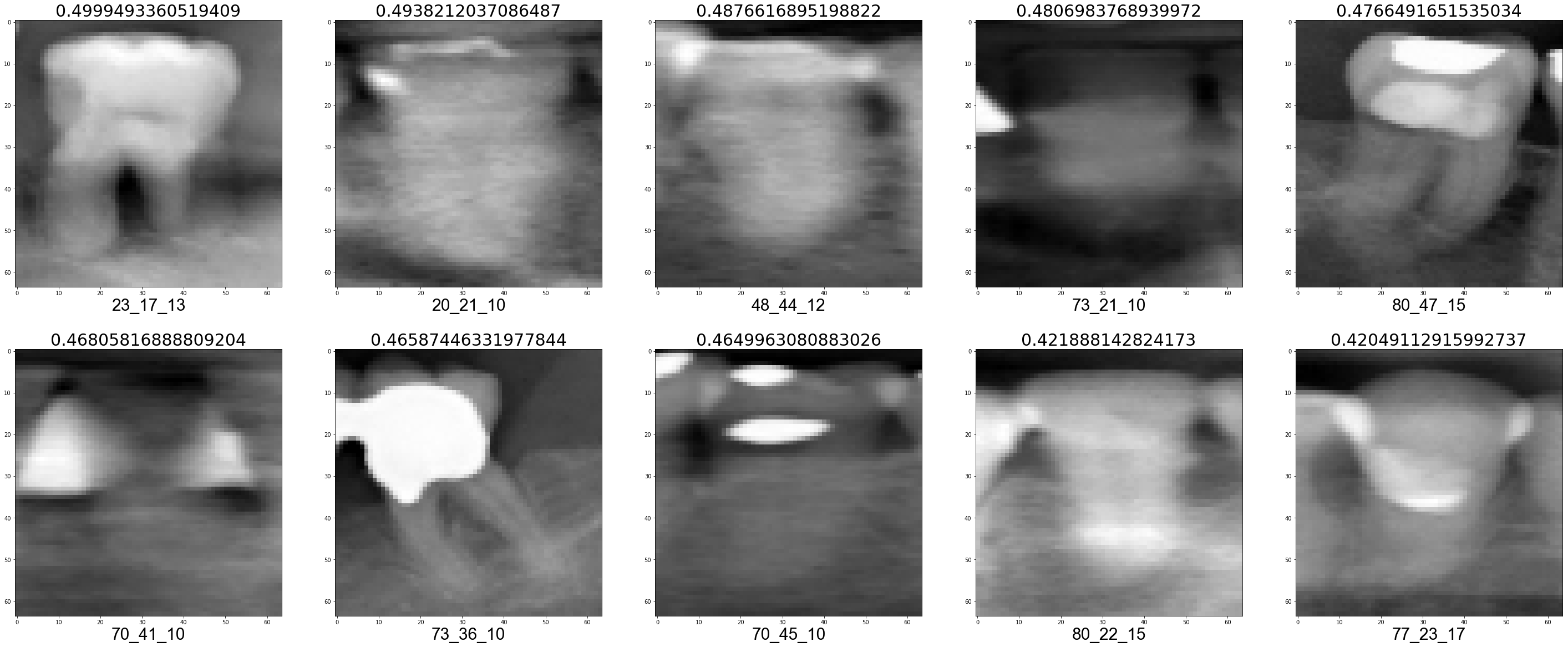


# Validation fold 4

#
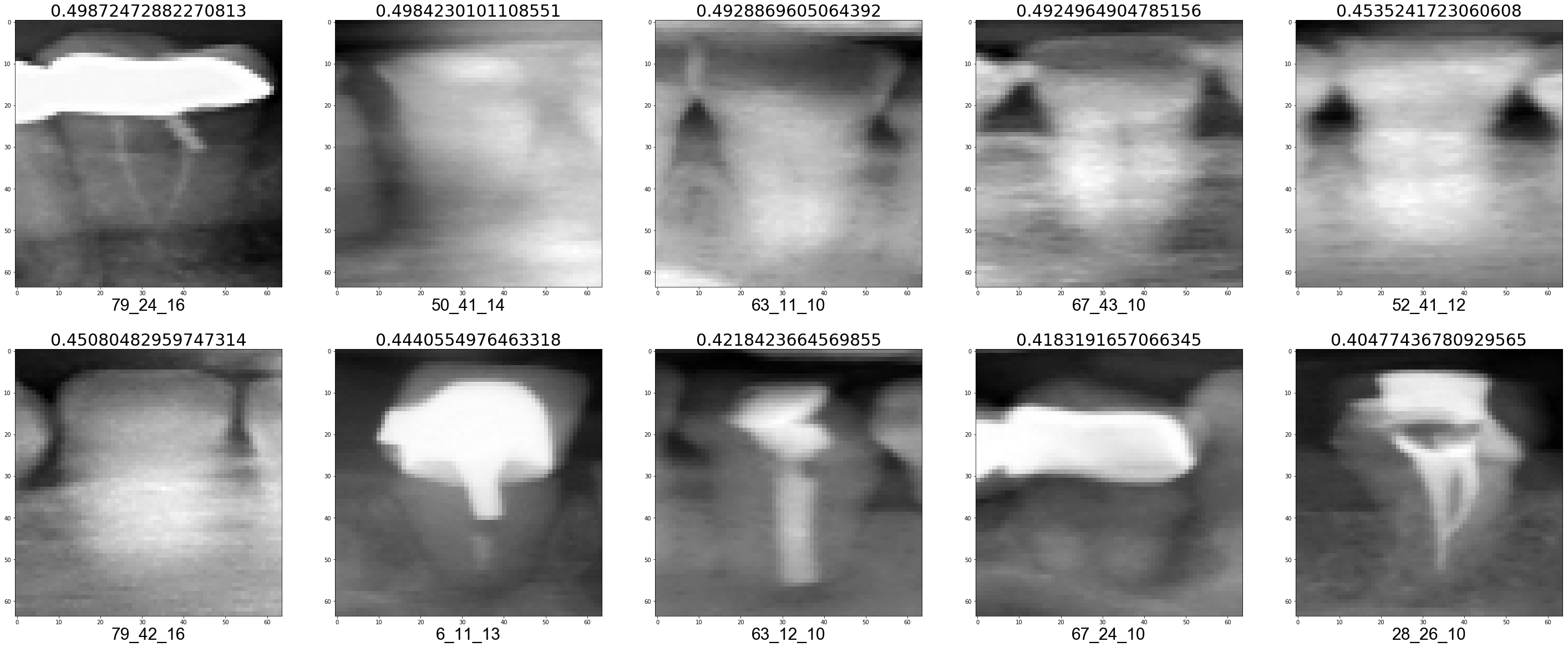


# Validation fold 5

#
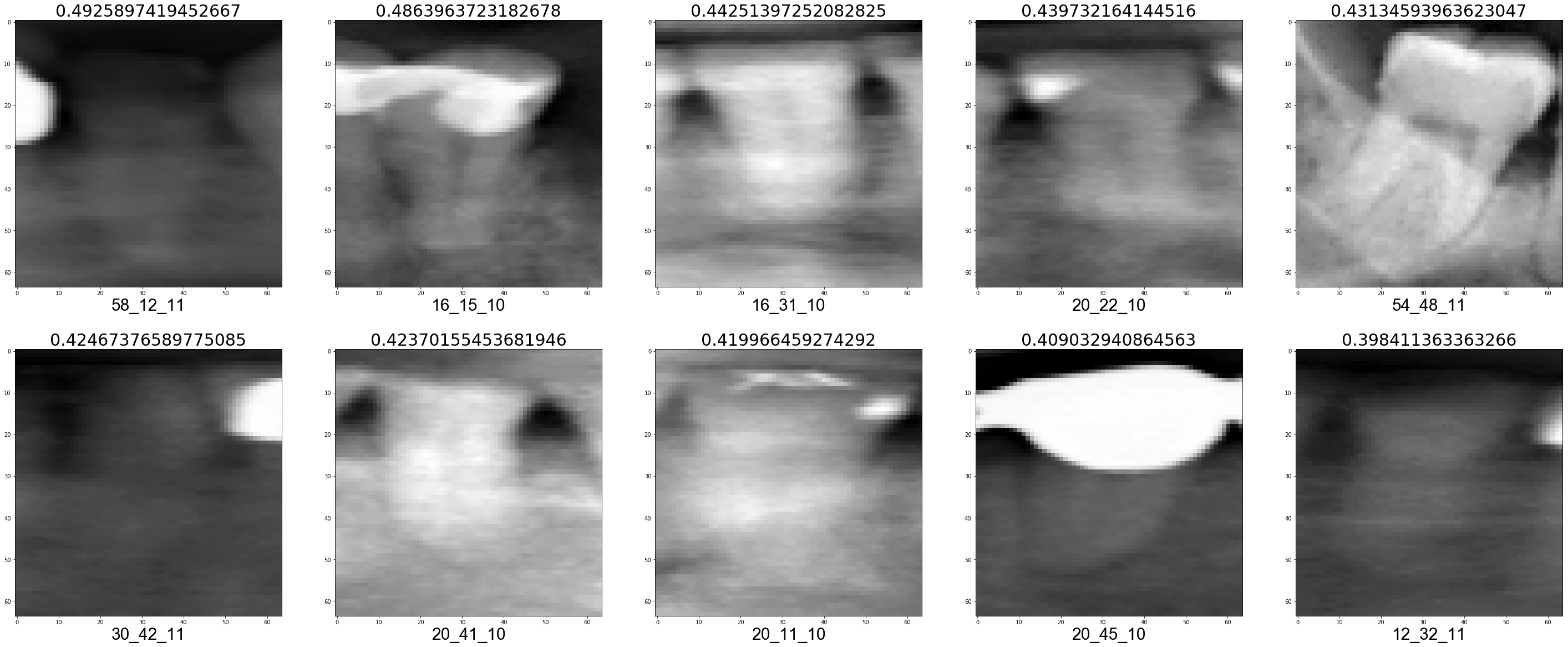


# Validation fold 6

#
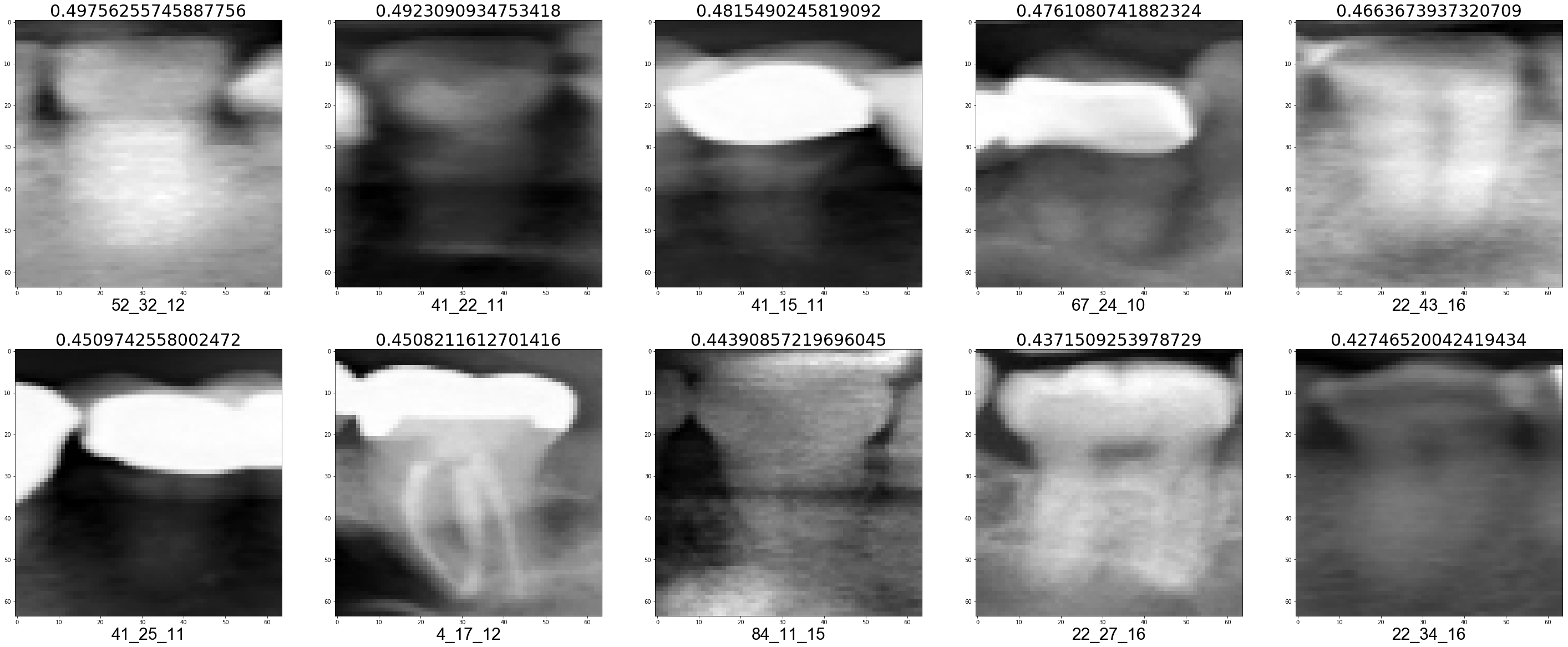


# Validation fold 7

#
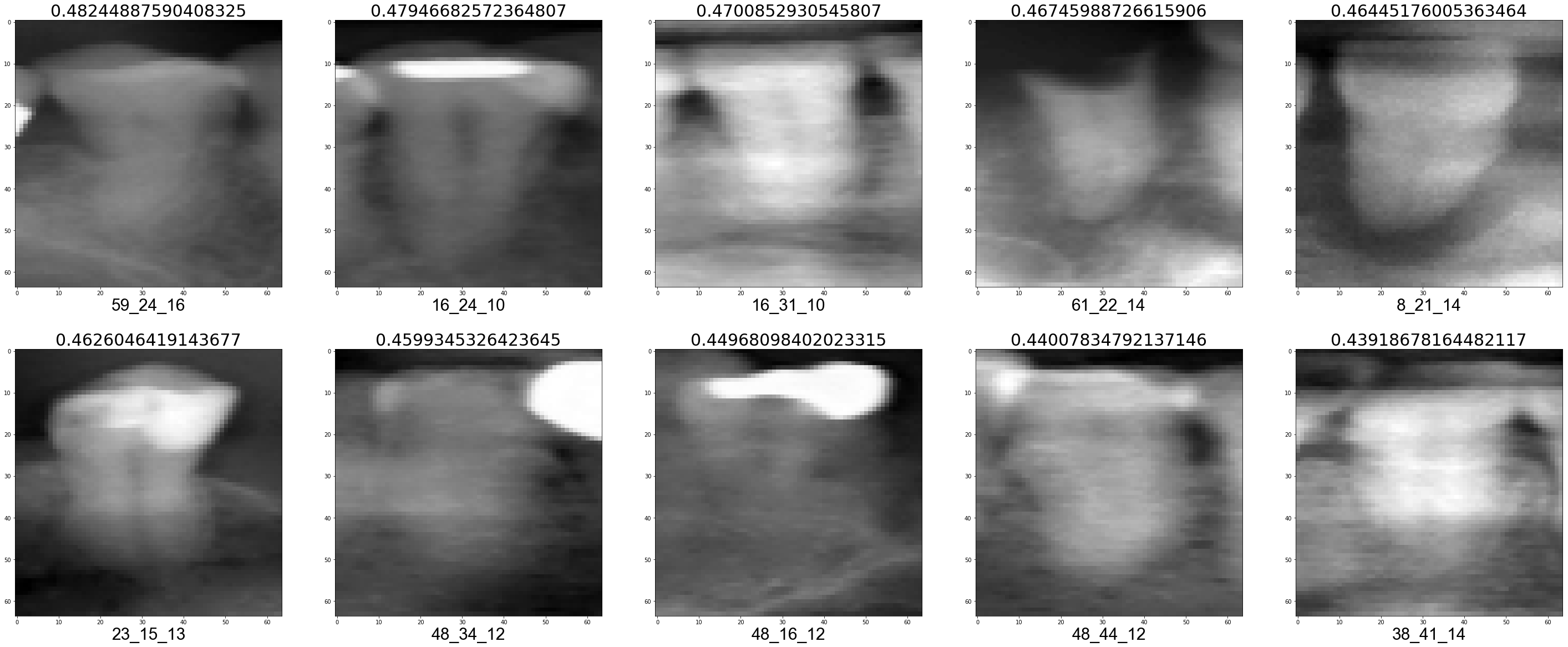


# Validation fold 8

#
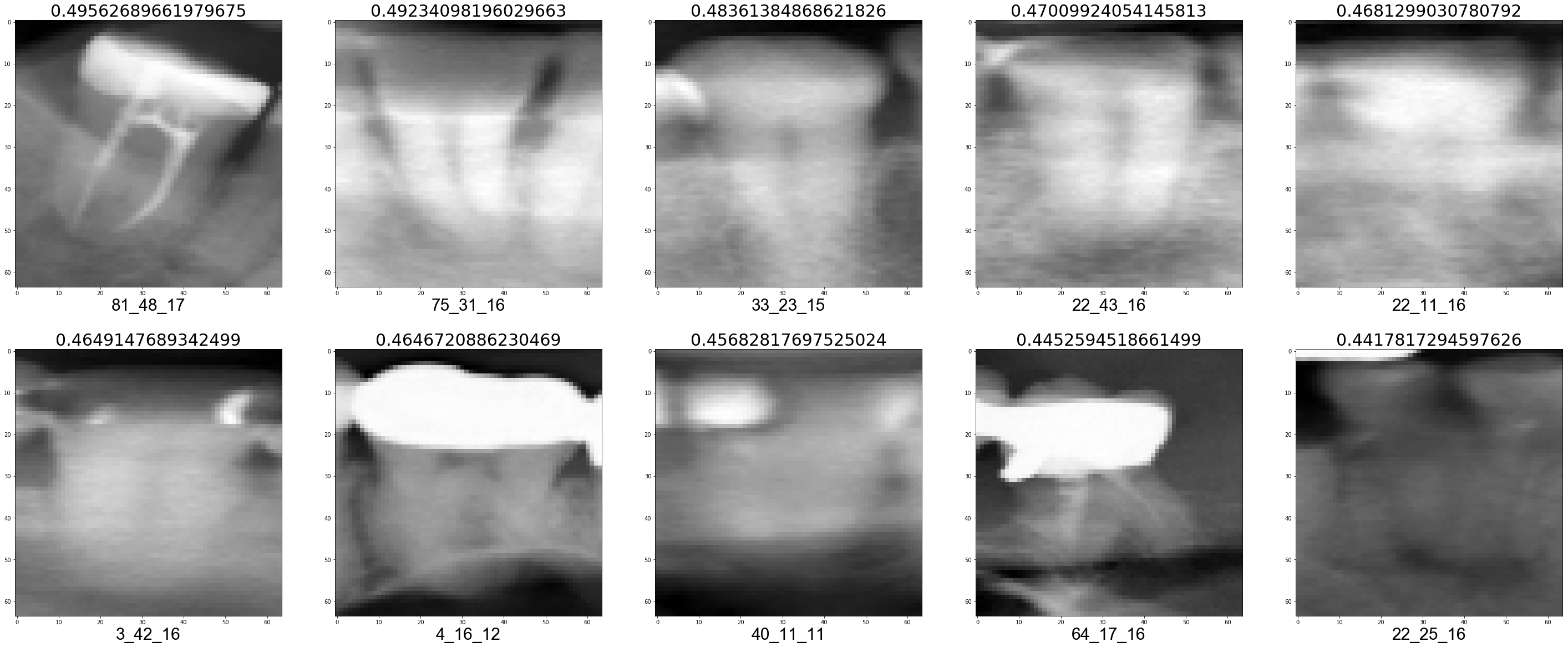


# Validation fold 9

#
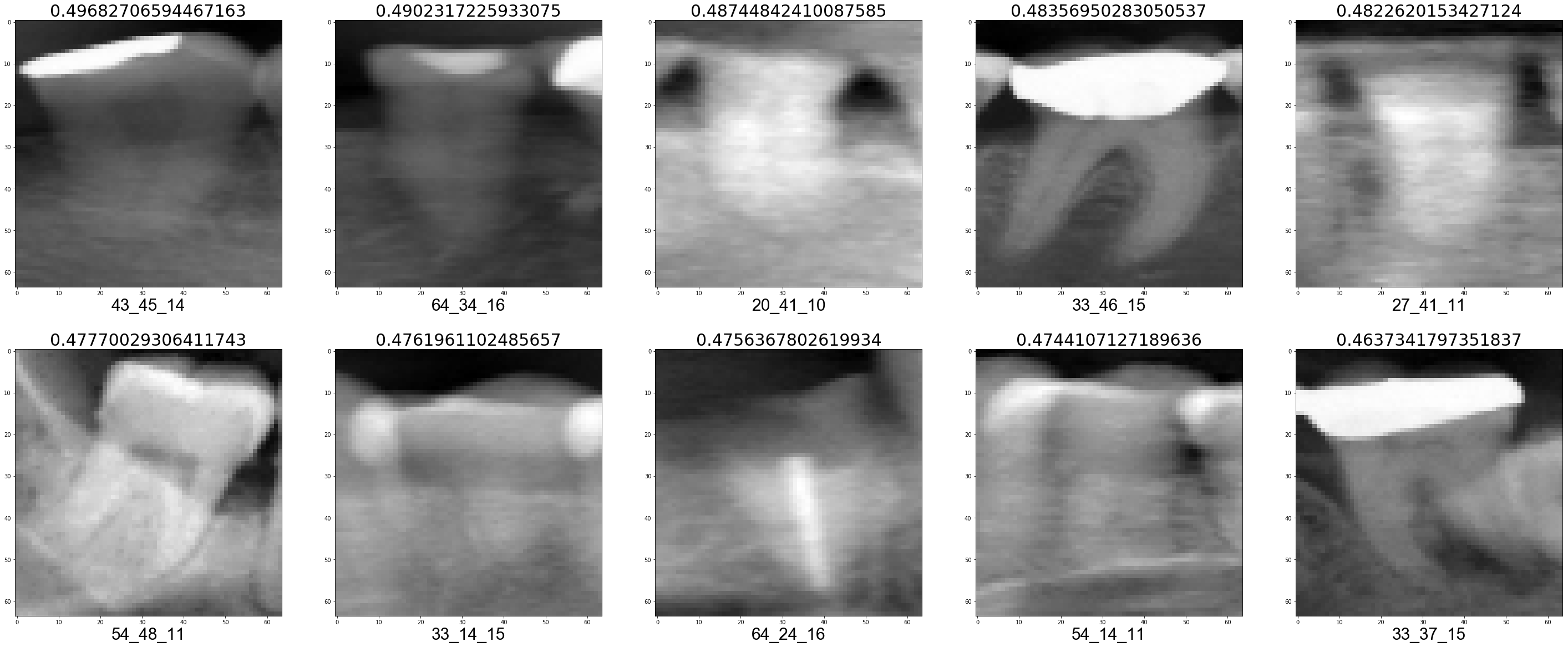


# Validation fold 10

#
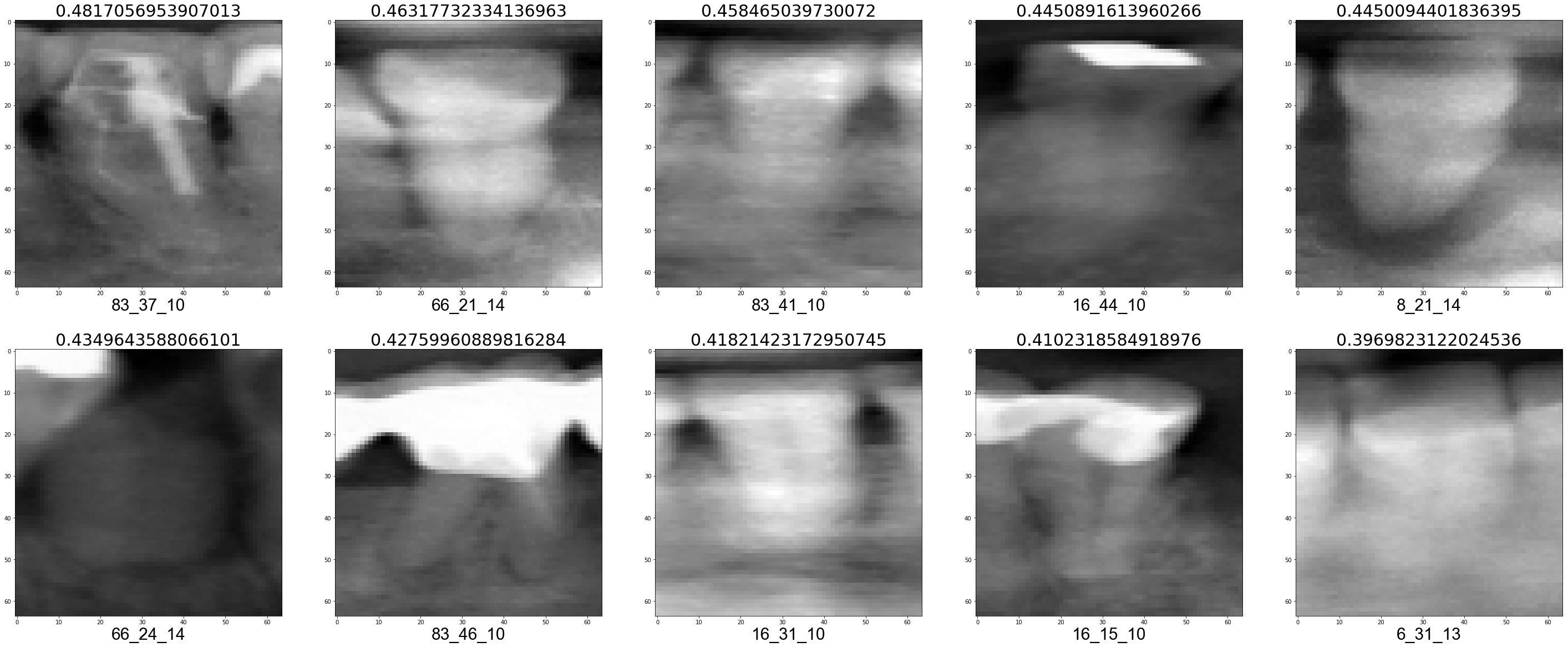


***Samples of included and excluded image segments***


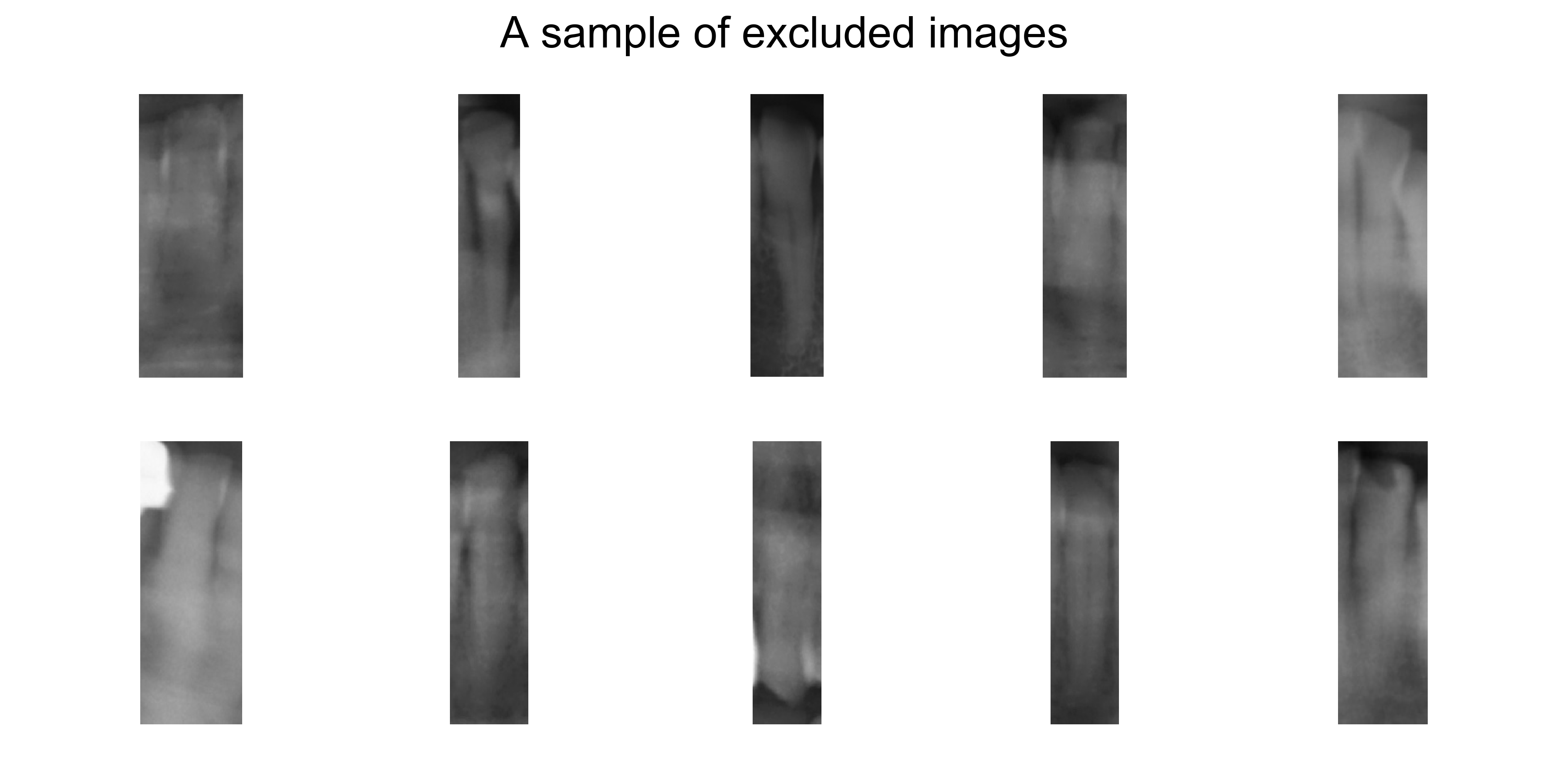

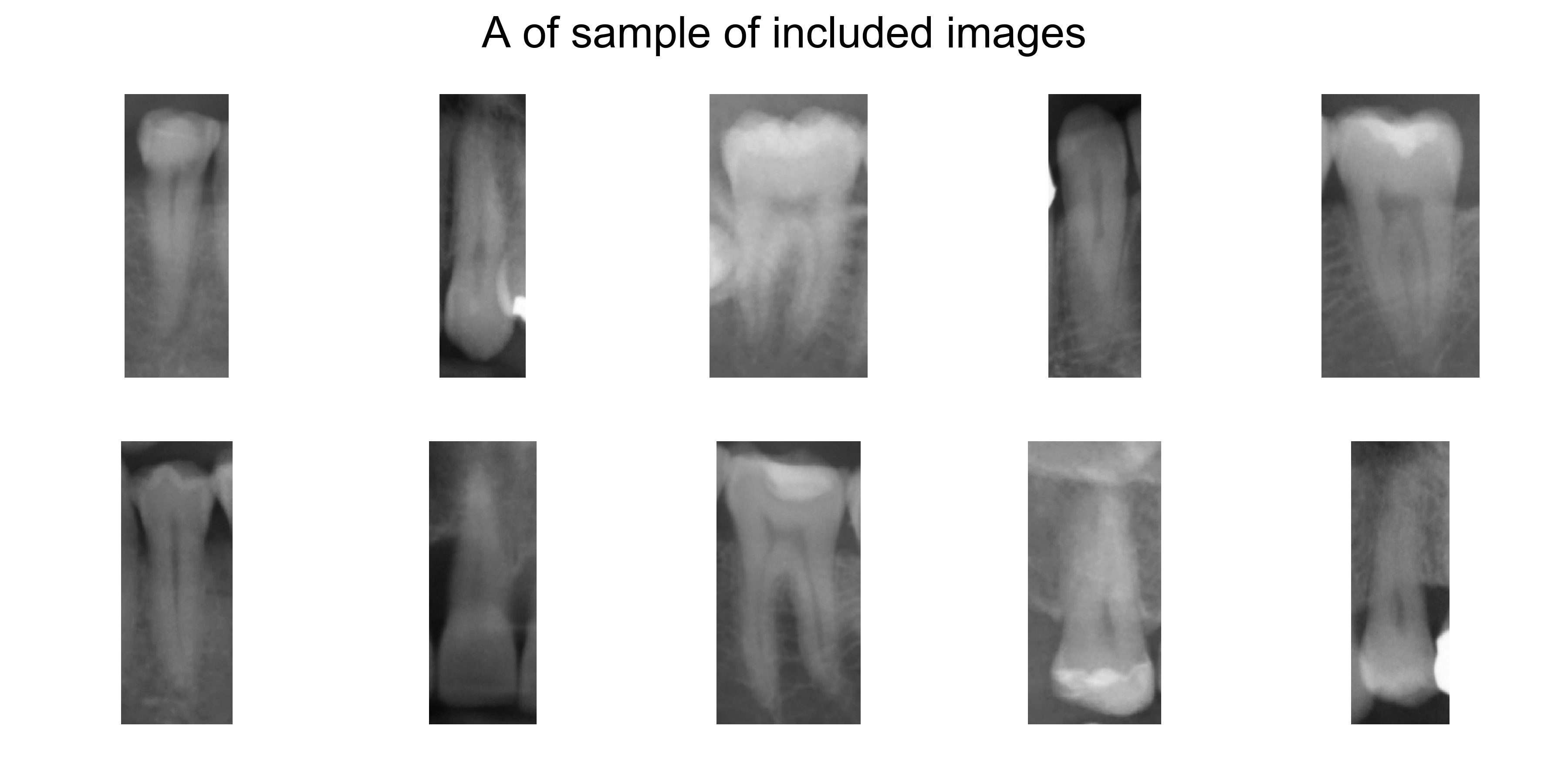


***References for the appendix***

Fleiss JL. 1971. Measuring nominal scale agreement among many raters. Psychol Bull. 76(5):378–382.

Gwet KL. 2008. Computing inter-rater reliability and its variance in the presence of high agreement. Br J Math Stat Psychol. 61(1):29–48.

Hussain Z, Gimenez F, Yi D, Rubin D. 2017. Differential Data Augmentation Techniques for Medical Imaging Classification Tasks. In: AMIA Annual Symposium Proceedings. p. 979.

Scott WA. 1955. Reliability of Content Analysis: The Case of Nominal Scale Coding. Public Opin Q. 19(3):321.

Sokolova M, Lapalme G. 2009. A systematic analysis of performance measures for classification tasks. Inf Process Manag. 45(4):427–437.
